# Possible biocontrol of bacterial blight in pomegranate using native endophytic *Bacillus* spp. under field conditions

**DOI:** 10.3389/fmicb.2024.1491124

**Published:** 2024-12-11

**Authors:** N. Manjunatha, Somnath S. Pokhare, Ruchi Agarrwal, Nripendra V. Singh, Jyotsana Sharma, Mallikarjun M. Harsur, Rajiv A. Marathe

**Affiliations:** ICAR-National Research Centre on Pomegranate, Solapur, India

**Keywords:** *Bacillus* spp., biological control agents, integrated disease management, *Punica granatum* L., volatile organic compounds, *Xanthomonas citri* pv. *punicae*

## Abstract

Bacterial blight in pomegranate, caused by *Xanthomonas citri* pv. *punicae* (Xcp), is one of the most devastating diseases, leading to substantial economic losses in pomegranate production. Methods for blight management in pomegranate production are scarce and not well established. To date, the major control strategy is targeting the pathogen with antibiotics and copper-based compounds. However, excessive use of antibiotics has resulted in the development of antibiotic resistance in the field population of Xcp. Hence, as a means of eco-friendly and sustainable management of bacterial blight, the use of native endophytes was investigated under field conditions in the current study. Endophytic bacteria were isolated from micro-propagated nodal explants of pomegranate and were identified as *Bacillus haynesii*, *B. tequilensis*, and *B. subtilis*. They were found to produce volatiles that inhibited Xcp growth during *in vitro* antibiosis assay. GC–MS-based volatile profiling revealed the presence of several bioactive compounds with reported antimicrobial activities. These endophytes (CFU of 10^8^/mL) were then spray-inoculated on leaves of 6-month-old pomegranate plants in the polyhouse. They were found to induce ROS-scavenging enzymes such as catalase and peroxidase. This alteration was a manifestation of host tissue colonization by the endophytes as ROS scavenging is one of the mechanisms by which endophytes colonize the host plants. Furthermore, two-season field trials with endophytes for blight control resulted in a reduction of disease index by 47–68%, which was considerably higher than the reduction due to the chemical immune modulator (2-bromo-2-nitro-1, 3-propanediol) currently being recommended for blight control. In addition, these endophytes also exhibited reduced sensitivity to this immune modulator; thus, the current study advocates the use of *B. haynesii*, *B. subtilis*, and *B. tequilensis* as biocontrol agents for bacterial blight of pomegranate either alone or as a part of integrated disease management.

## 1 Introduction

Pomegranate (*Punica granatum* L.), also known as the “Fruit of Paradise,” has edible red arils that are highly rich in anti-oxidants and have proven health benefits (Seeram et al., 2006; Viuda-Martos et al., 2013). These benefits include reduction of inflammation, muscle damage, and an increase of platelets blood levels to name a few. In addition to edible parts, non-edible parts of the tree can also be used commercially owing to their several beneficial properties, for example, peel powder can be used as a food preservative (Lacivita et al., 2021; Giri et al., 2024). Owing to its innumerable benefits and uses, the global pomegranate market is expected to expand to USD 33.86 (by 2028) from USD 24.8 billion (2021). Furthermore, due to its adaptability to a wide range of weather and soil conditions, fresh pomegranate fruit is available in India all throughout the year. Therefore, India has become one of the global leaders in pomegranate production. Currently, India ranks seventh in the production of pomegranate in the world with a total area under cultivation of approximately 275,500 hectares, producing 3,215,000 metric tons of pomegranate fruits (DA& FW, GoI, 2023). Notably, in 2022–2023, 62,280 metric tons of pomegranates worth USD 58.36 million were exported from India (APEDA, 2024). Consequently, pomegranate plantation has played a very significant role in improving the socio-economic scenario of small landholders (2.5 million), particularly in the Indian states of Maharashtra, Karnataka, Gujarat, Rajasthan, and Andhra Pradesh. However, as with any other crop, pomegranate production is threatened by several pests and diseases due to the unavailability of any resistant germplasm.

One of the most devastating diseases of pomegranate is bacterial blight caused by *Xanthomonas axonopodis* pv. *punicae* (Syn = *Xanthomonas citri* pv. *punicae*: Xcp), which was first reported in India in the 1950s (Hingorani and Mehta, 1952). The bacterial blight symptoms mostly appear on the fruits and leaves, sometimes the stem too, leading to damage to the whole plant and economic losses to the grower (Sharma et al., 2017). In India, bacterial blight, also called oily spot, can result in 60–80% and in some cases 100% losses in fruit yield (Sharma et al., 2022a). Until now, the main strategy for bacterial blight control in pomegranate production relied upon the application of antibiotics and copper-based chemical compounds (Benagi et al., 2012; Sharma et al., 2022b). Extensive use of antibiotics in agriculture may, however, cause a reduction in the efficacy of antibiotics by facilitating the emergence of antibiotic resistance among the pathogens. For instance, *Erwinia amylovora* has been reported to have developed antimicrobial resistance in a number of geographic locations where antibiotics have been used indiscriminately in apple and pear orchards (Tancos et al., 2016). The impact of streptomycin-resistant *E. amylovora* in orchards remains a subject of ongoing scientific debate (Förster et al., 2015). Recently, reduced sensitivity to antibiotics and copper compounds in the field populations of pomegranate bacterial blight pathogen has also been reported from India (Krishna et al., 2020; Sharma et al., 2022a). Moreover, the majority of antibiotics are non-specific, meaning they not only act against the target pathogen but may also affect other beneficial bacteria naturally present in or on the plants (Daisley et al., 2022). Consequently, the Government of India issued a draft gazette notification on “Prohibition of Streptocycline (Streptomycin sulfate 90% w/w + Tetracycline hydrochloride 10% w/w) in agriculture and horticulture” dated 17 December 2021, released by the Ministry of Agriculture and Farmer’s Welfare. The notification imposes a ban on the manufacture and import of this product, w.e.f: with effect from 1 February 2022, with the ban on its use set to take effect from 1 January 2024. This gazette was brought out by the government because of the growing concerns over the risk of the development of resistance to these antibiotics in human beings and animals. Thus, the development of efficient and eco-friendly technologies focused on the elimination or reduction of the application of antibiotics in agriculture is highly desirable. In addition, globally the export of residue-free fruits is highly promoted, therefore providing an impetus to find new alternatives for organic production of pomegranate.

One such promising approach is the utilization of microbes for the biological control of pathogens (Berg and Hallmann, 2006; Morales-Cedeño et al., 2021). Biocontrol agents (BCA) can mediate plant protection through direct mechanisms such as the production of secondary metabolites (Pretali et al., 2016) or hydrolytic enzymes that have antagonistic effects on the growth and survival of plant pathogens (Oukala et al., 2021); or indirect mechanism of defense involving the phenomenon of induced systemic resistance (ISR) (Wei et al., 1991), through jasmonic acid/ethylene pathway (Kannojia et al., 2019). ISR induction occurs when microbe-associated molecular patterns (MAMPs) of the BCAs are recognized by the plant/host and a defense response is mounted (Pieterse et al., 2014). This type of resistance is durable because the chances of the development of resistance in a pathogen against this type of resistance are very low as the pathogen does not directly interact with the BCAs or the resistance-stimulating agent (Romanazzi et al., 2016). Consequently, more recently attention has been focused on the beneficial aspects of endophytes and the application of these endophytes as BCAs (Backman and Sikora, 2008; Strobel, 2018; Collinge et al., 2022). Endophytes, derived from the Greek words “endo” meaning “inside” and “phyte” meaning “plant,” are symbiotic groups of microorganisms that colonize the internal tissues of plants asymptomatically (Nair and Padmavathy, 2014; Santoyo et al., 2016). These endophytes, apart from being effective BCAs, also have plant growth-promoting (PGP) activities through (i) production of enzyme 1-Aminocyclopropane-1-carboxylase (ACC) deaminase, which is the precursor to plant growth regulator ethylene, hence reducing the levels of ethylene; (ii) nutrient acquisition such as nitrogen, phosphate, potassium, and iron; and (iii) synthesis of phytohormones such as auxin, cytokinin, and GA (Santoyo et al., 2016). A significant range of endophytic bacteria and fungi have been isolated from a variety of plants and shown to have a number of positive effects on the host plant (Bacon and Hinton, 2007; Manjunatha et al., 2022; Salvi et al., 2022). Such benefits embrace the lessening of chemical fertilizer usage, the enhancement of soil quality, the conservation of the environment, and sustainable agriculture. The well-studied and most abundant organisms isolated from plant tissues belong to Actinobacteria, Proteobacteria, and Firmicutes and include members of the genera *Streptomyces, Pseudomonas, Azoarcus, Enterobacter, Burkholderia, Stenotrophomonas*, and *Bacillus* (Malfanova et al., 2013; López et al., 2018).

Horticultural crops, including vegetables and fruits, harbor endophytic bacteria (*Pseudomonas* and *Bacillus*), and many of them have been reported to possess antimicrobial properties against a wide range of pathogens (Ray et al., 2016; Pavithra et al., 2021). Despite adequate knowledge of the diversity of bacterial endophytes in fruit crops, studies on their biocontrol potential against pomegranate disease are scarce. In one of the studies, the antagonistic activity of endophytic bacteria or fungi isolated from different wild genotypes or cultivars of pomegranate was tested against Xcp (Karn et al., 2022), and in another study, the endophytes were isolated from pomegranate roots and tested against wilt causing pathogen (Maruti and Sriram, 2021). However, both these studies were preliminary as they focused only on the *in vitro* antagonism. More recently, *in vitro* and *in planta* antagonistic effects of bacterial endophytes against Xcp, the most dreaded bacterial pathogen of pomegranate, were reported (Singh et al., 2023). It was the first report of endophytes isolated from micro-propagated pomegranate plants, and the isolates could inhibit the pathogen *in vitro* and under polyhouse conditions. However, there are still no reports of utilization of endophytes for biocontrol of *Xanthomonas citri* pv. *punicae* under field conditions. Moreover, none of the abovementioned studies deciphered the probable mechanism by which the endophytes exert their antagonistic effect on the pathogen.

Therefore, the current study was undertaken to decipher the probable mechanisms behind the biocontrol action of endophytes and to evaluate their effectiveness against *X. citri* pv*. punicae* under field conditions. Specifically, the study aimed to: (i) isolate and identify endophytes with potential biocontrol activity against the bacterial blight pathogen; (ii) assess the antagonistic activity of volatile compounds produced by these endophytes against the pathogen using an *in vitro* approach, and profile the volatile organic compounds (VOCs) through GC–MS to identify the antimicrobial compounds; (iii) evaluate the alteration in the host’s biochemical response upon endophyte inoculation and confirm *in vivo* host colonization by the endophytes; (iv) conduct on-field trials to assess the effectiveness of endophytic bacteria against bacterial blight, as well as test the sensitivity of endophytic bacteria to bactericides and copper compounds (used in current practices for blight management) *in vitro*. Although the results of the present study advocate the use of potential bacterial endophytes for biocontrol of pomegranate bacterial blight under field conditions, it will be important to standardize the mode of application of these endophytes in the future for better results. In addition, there is a need to evaluate the scalability of this endophyte technology for commercial use and its long-term impact on pomegranate production.

## 2 Materials and methods

### 2.1 Isolation and selection of endophytic bacteria as biocontrol agent

Bacterial endophytes were isolated from tissue-cultured (TC series) pomegranate plants of Bhagwa variety in our previous study (Singh et al., 2023). Isolated pure colonies were then picked up and maintained as pure culture until further use. The isolates were tested for their antagonistic activity against the bacterial blight pathogen (*Xanthomonas citri* pv. *punicae*: Xcp) using *in vitro* dual culture assays (Supplementary Figure S1A) and under polyhouse conditions. On the basis of the screening results, three effective isolates were selected for further studies in the present investigation. These isolates exhibited equal to or more than 50% inhibition of pathogen growth *in vitro*, and under polyhouse conditions, they either completely checked the incidence of bacterial blight or reduced the severity of the symptoms. Pure cultures of these isolates have been deposited at a culture repository under the National Biodiversity Act, 2002 with accession numbers NAIMCC-B-03178 to NAIMCC-B-03180.

### 2.2 Molecular identification of potent endophytic bacteria

Effective bacterial endophytes were identified at the molecular level based on a partial 16S rRNA gene sequence. Cultures grown in nutrient glucose broth (NGB) for 24 h with shaking at 120 rpm at 28 ± 1°C were used for genomic DNA (gDNA) isolation using HiPurATM Bacterial Genomic DNA Purification kit (HiMedia) as per the manufacturer’s instructions. For each isolate, PCR was performed using good quality gDNA and amplified using universal primers for 16 s rRNA region (F-AGAGTTTGATCCTGGCTCAG and R-GGTTACCTTGTTACGACTT) (Patel, 2001) with initial denaturation at 94°C for 4 min, 35 cycles of denaturation at 94°C for 30s, annealing at 50°C for 30s, and extension at 72°C for 1 min, followed by final extension at 72°C for 8 min (Manjunatha et al., 2023).

### 2.3 Phylogenetic analysis of potent endophytic bacteria

Phylogenetic analysis was carried out to confirm the identity and understand the genetic relatedness of the effective isolates with other bacteria belonging to the same genus and species. The 16 s rRNA region amplified from bacterial endophytes and deposited at the GenBank NCBI with accession numbers ON629736, KY575578, and KY575582, along with reference sequences downloaded from the database was aligned using the MUSCLE algorithm. BLASTn search, limiting the search to type material, was performed, and the reference sequence with the maximum similarity was included in the phylogenetic analysis. Neighbor-joining trees were drawn, evolutionary distances were computed using the Tamura-Nei method, 1,000 bootstrap replications were performed, and the consensus tree was rooted to *Paenibacillus polymyxa* and reported. Since *Paenibacillus polymyxa* has been found to be phylogenetically distinct from *Bacillus* spp. (Ash et al., 1993), it was used as an out-group in the current study. This analysis involved 20 nucleotide sequences including three from the isolates examined in the current study. All ambiguous positions were removed for each sequence pair (pairwise deletion option) after which there were a total of 1,496 positions in the final dataset. Phylogenetic analyses were conducted in MEGA11 (Tamura et al., 2021).

### 2.4 Study of antimicrobial properties of the potent endophytic bacteria

#### 2.4.1 Antibiosis test for production of inhibitory volatile compounds

Inhibitory effects of volatiles secreted by endophytic *Bacillus* spp. on the growth of Xcp were assayed using a method that prevented direct contact between the two microbes, thereby excluding any contact-dependent inhibitory effects of the endophytic bacteria on the pathogen. In this method, adopted from the study by Dennis and Webster (1971), two nutrient glucose agar (NGA) plates were used in which one of the plates was inoculated with the pathogen and kept on top of the other plate that was inoculated with the endophyte (Supplementary Figure S1B). Volatiles would be emitted by the endophytes in the upward direction; hence, the endophyte plate was kept below, and the pathogen plate was kept on top. For the control setup, one of the plates was inoculated with the pathogen, and the other plate contained un-inoculated NGA only such that if any effect is produced by the media components, it will be visible in the control plates. The plates were sealed with parafilm and incubated at 28 ± 1°C, making sure that the pathogen-containing plate was on top of the endophyte-containing plate and the growth of Xcp was monitored and recorded. The growth of Xcp was monitored in terms of (i) zone of inhibition in the area just above the endophyte-inoculated area, (ii) reduction in cells/colonies, and (iii) reduction in yellow pigment production.

#### 2.4.2 Identification of antimicrobial compounds through gas chromatography–mass spectrometry

Solvent (methanol and ethyl acetate) extracts of *Bacillus* culture were subjected to GC–MS to identify their volatile organic compound profile. In brief, bacterial isolates were inoculated in nutrient glucose broth (NGB) and incubated at 28 ± 1°C for 24–48 h on a shaker incubator (120 rpm). When the OD_600_ was 1.5, then 1:10^5^ dilution was performed to obtain an initial inoculum of 200–300 CFU per 10 μL, and 10 μL of the culture was added in 50 mL NGB and incubated again after which volatile collection was performed. In brief, the culture flask was placed with a volatile collection assembly customized to have two lateral openings in an aseptic chamber (Nagrale et al., 2022). Volatile collection was carried out for 4 h, and volatiles were consecutively eluted in the desired solvent (methanol or ethyl acetate) in a final volume of 250 μL (Nagrale et al., 2022). GC–MS-QP2020 (Shimadzu) equipped with a DB5-MS column was used for volatile separation and spectrum analysis. The GC–MS columns and settings were as detailed in Nagrale et al. (2022). Identification of metabolites was performed based on mass spectra similarity with library spectra available at the National Institute of Standards and Technology (NIST-2014 version).

### 2.5 *In planta* experiments for the evaluation of host biochemical response upon endophyte inoculation

The effective endophytes were exogenously sprayed onto pomegranate plants, under polyhouse conditions. In brief, endophytes were inoculated in NGB and kept on a shaker incubator at 28°C for 24–48 h. The bacterial suspension was then diluted to 10^8^ cells per ml (OD_600_ nm = 0.4), of which approximately 25 mL solution was sprayed on each plant. Three biological replicates were used for each treatment, i.e., TC-4, TC-6, and TC-310 inoculated plants as well as the control (not inoculated with any endophyte). Host biochemical response was observed by measuring (i) the activity of enzymes involved in the anti-oxidative defense system, namely catalase, peroxidase (POD), and superoxide dismutase (SOD), and (ii) total phenolic content (TPC) in the host.

#### 2.5.1 Total phenolic content estimation

Total phenols were extracted by crushing 0.5 g of leaf tissue in 5 mL of 30% ethanol. The sample was centrifuged at 10,000 rpm for 20 min, and the supernatant was collected. Five milliliters of 30% ethanol was added to 1 mL supernatant. The solvent was evaporated by incubating in a hot air oven at 60°C for 3 h. The sample was resuspended in water and used for TPC estimation (Waterhouse, 2002). TPC was measured against a standard curve of gallic acid with absorption at 765 nm, and gallic acid equivalents (GAEs)/g of dry plant material were calculated for control (un-inoculated) and treatment (endophyte-inoculated).

#### 2.5.2 Enzyme extraction and activity estimation

Enzymes were extracted by crushing 0.5 g leaf tissue in 10 mL of 0.1% trichloroacetic acid (TCA). The sample was centrifuged at 10,000 rpm for 20 min, and the supernatant was collected as a source of enzymes.

Catalase activity was measured using a hydrogen peroxide (H_2_O_2_) standard curve with absorption at 240 nm (Aebi, 1984). One unit of enzyme activity was defined as the amount of enzyme required to decompose 1 μmole of H_2_O_2_ per min. Total peroxidase activity (POD) was measured using guaiacol as a standard with absorption at 470 nm (Chance and Maehly, 1955). One unit of enzyme activity was defined as the amount of enzyme required to produce 1 μmole of guaiacol per min.

Superoxide dismutase (SOD) activity was measured following the nitroblue tetrazolium (NBT) reduction method (Dhindsa et al., 1981). The reaction mixture (3 mL) contained 50 mM phosphate buffer, pH 7.8, 26 mM methionine, 20 μM riboflavin, 750 μM NBT, and 1 μM EDTA. After adding enzyme solution (0.1%) and distilled water, the reaction was allowed to run for 15 min under 4,000 lx light. The absorbance was recorded at 560 nm, and one unit of enzyme activity was defined as the amount of enzyme that reduced the absorbance by 50% of the absorbance of no enzyme control.

Fold change, in enzyme activity or accumulation of TPC, between endophyte-inoculated (treatment) and un-inoculated (control) plants was calculated and finally reported.

### 2.6 Host colonization by the endophytes

The host colonization pattern of the endophytes was checked *in vivo*. In brief, pomegranate plants were sprayed with endophyte culture [CFU of 10^8^/mL (absorbance at 600 nm ≈ 0.4)] in a polyhouse. Then, 25 mL of endophyte culture was sprayed manually on each plant, and plants that were not inoculated with this endophyte served as control. Leaves from inoculated plants were excised at 24-h intervals, and periodic isolations were taken to check the colonization pattern of the endophytes. The leaf tissues were cut into small pieces and surface-sterilized with sodium hypochlorite (1%) for 30 s and ethanol (70%) for 30 s followed by three rinses with sterile distilled water (Anjum and Chandra, 2015). The sterile leaf tissues were then placed onto nutrient glucose agar media containing Petri dishes. Water after the last rinse was also plated to confirm the endophytic nature of the organism. Isolations in the same way were also taken from control plants. The bacterial colonies obtained in treated plants that were different from those obtained in control plants were only considered; then, these were checked for their morphological similarities with TC-310 that was sprayed onto plants. On these two bases, the frequency of endophyte colonies was measured, and percent recovery was calculated. Furthermore, 16S sequencing was performed to confirm the isolation of endophytes.

### 2.7 Bio-efficacy of potent endophytic bacteria under field conditions

The effective endophytes were utilized for field trials at research farms of the ICAR-National Research Centre on Pomegranate (Hiraj, Solapur, Maharashtra, India) and compared to the performance of the immune modulator (2-bromo-2-nitro-1, 3-propanediol) and water control. The research plot (H-22) was a 5-year-old (in 2022) pomegranate orchard cv. Bhagwa with a spacing of 4.5 m × 3.0 m. This plot was selected for the study and is dedicated to all ongoing bacterial blight-related studies at the center. No bacterial control sprays were applied throughout the trial period, up to harvesting. The season selected for the study was *Mrig* bahar (Rainy season), during which there is maximum incidence of bacterial blight naturally due to conducive weather conditions. The age of the plants in the research orchard was 5 years at the time of fruit regulation during the 2022 season trials.

A randomized block design experiment was carried out with five treatments, three replicates, and two plants per replicate. The treatments included T1: TC-4 (*B. haynesii*), T2: TC-6 (*B. subtilis*), T3: TC-310 (*B. tequilensis*), T4: immunomodulator (bactronol), and T5: control (water). Bacterial endophytes were grown in NGB overnight. The next day, the culture was used as a primary culture to inoculate larger volumes of NGB to obtain a final volume of the culture (6 L) with a total CFU of 10^8^/mL or higher (absorbance at 600 nm ≈ 0.4). This culture was sprayed onto plants, with 1 L of the culture or chemical solution sprayed per plant, and 0.3 mL of sticker was added to all solutions. In the case of the control, 1 L of water with 0.3 mL of sticker was sprayed. A total of five sprays, at an interval of 5 days each, were performed after fruit setting and when blight Percent Disease Index (PDI) reached 5% in at least one of the treatments. For observation of disease incidence and severity, five branches with maximum fruits on each branch were labeled, and data were recorded for only these branches. Hence, for one plant, the data were mean of five branches. Observations for disease incidence and severity on leaves and fruits were recorded weekly, and the percent disease index (PDI) was calculated using the formula given below (Sharma et al., 2017). To confirm the pathogen, isolation of Xcp was performed in the laboratory from infected leaves. Such field trials were conducted for 2 consecutive years (2022 and 2023) during *Mrig bahar* (May–June crop; second or monsoon flowering)


$$PDI=\left[\frac{\sum \text{Number of observation}\times \text{Severity grade}}{\text{Total observations}\times \text{Maximum grade}}\right]\times 100$$

### 2.8 Chemical sensitivity test

The selected endophytes were tested for their sensitivity to commercially available bactericides and other chemicals using a good diffusion assay (Holder and Boyce, 1994). These chemicals included the immune modulator: 2-bromo-2-nitro-1, 3-propanediol (0.5 g/L); and other copper-based compounds: copper hydroxide (2 g/L) and copper oxychloride (2 g/L). Their doses were chosen as per the recommendation of the center to pomegranate farmers for field application during bacterial blight management. In brief, culture suspension of bacterial colony with a CFU of 10^8^/mL was made in sterile distilled water and spread on NGA plates. A well of 6 mm was dug in the middle of the plate using a sterile cork borer, and the required volume (100 μL) of the test chemical was added to the well. The clear zone around the well, which was devoid of any bacterial growth, i.e., the zone of inhibition (ZOI), was measured using zone scales (HiMedia) after 24 h of growth at 28 ± 1°C in a BOD incubator. Percent inhibition (PI) in the growth of endophytes on the chemical was calculated by the following formula and plotted.


$$\text{PI}=100-\left[\frac{\begin{array}{l} \text{Diameter of colony}\;\text{on}\;\text{control plate}-\\ \text{Zone of inhibition}\;\text{on}\;\text{treatment plate} \end{array}}{\text{Diameter of colony}\;\text{on}\;\text{control plate}}\right]\times 100$$

### 2.9 Statistical analysis

All experiments were carried out with a minimum of three replicates with appropriate controls. For biochemical response, a *t*-test was carried out to determine whether the difference (fold change) between treatment and control was statistically significant at a *p*-value of < 0.05. For polyhouse experiments, five plants were taken per treatment considering each plant as one replicate, and the experiment design was completely randomized design (CRD). For field studies, three replicates per treatment were taken with two plants per replicate, and the experiment design was randomized block design (RBD). Statistical analysis was performed using WASP 2.0^1^ (Jangam and Wadekar, 2004), and the critical difference was expressed at a *p*-value of ≤ 0.05.

## 3 Results

### 3.1 Isolation and selection of endophytic bacteria

Microbes isolated from plant endosphere, inhabiting the internal tissues in a symptomless manner, are known as endophytes. Such microbes can also be isolated from tissue-cultured plants and may have a diverse impact on the growth of hosts. In our previous study (Singh et al., 2023), we reported, for the first time, the isolation of endophytes from nodal segments of micro-propagated pomegranate plants. These endophytes (TC series) along with endophytes isolated from field-grown pomegranate plants (EB series) were tested for their antagonistic effects on the growth of bacterial pathogen *Xanthomonas citri* pv. *punicae* (Xcp) *in vitro* and *in planta*. We observed higher inhibitory effects were shown by TC series endophytes as compared to EB series endophytes both *in vitro* and *in planta*, for example, three endophytes, namely TC-4, TC-6, and TC-310 showed equal to or more than 50% inhibition of Xcp *in vitro*, while none of the EB series endophytes showed equal to or more than 50% inhibition. During *in planta* antagonistic assays, the endophytes were applied in prophylactic mode, i.e., sprayed prior to pathogen challenge inoculation at an interval of 8 days. Plants inoculated with TC-4 or TC-310 did not show any symptoms, indicating that these endophytes had controlled blight incidence completely, while plants inoculated with TC-6 exhibited lower disease incidence and severity. Based on these results, it was deduced that these three isolates showed varying mechanisms by which pathogen’s growth was inhibited, and therefore in the current study, these isolates and their inhibition mechanisms were investigated further.

### 3.2 Molecular characterization and identification of potent endophytic bacteria

**Figure 1:**
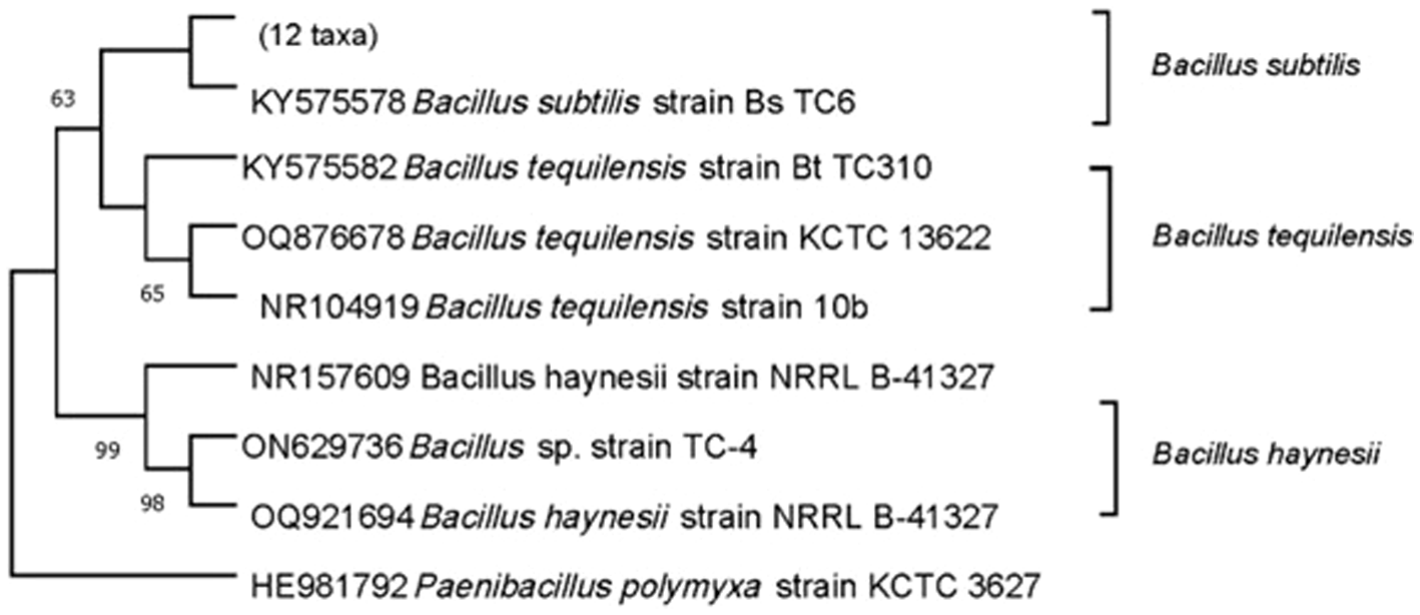
Molecular characterization and identification of endophytic bacteria used as biological agents in the study. Neighbor-joining tree depicting the phylogenetic relationship between effective endophytes (TC-4, TC-6, and TC-310) and type strains’ sequences retrieved from the NCBI database. The Tamura-Nei method was used as the best model for constructing the phylogenetic tree with 1,000 replicates. The tree shown represents the bootstrap consensus tree inferred from 1,000 replicates, and numeric values on the branches indicate bootstrap support values in percentage. Branches corresponding to partitions reproduced in less than 50% bootstrap replicates are collapsed. *Paenibacillus polymyxa* was used as an out-group.

To understand the nature of the antagonistic mechanism, it is important to first identify the bioagent. In the current study, bacterial endophytes were identified based on BLASTn homology search of 16S rDNA sequence followed by phylogenetic analysis. When we did BLASTn for TC-310 (KY575582), limiting the search to type material, the maximum similarity was obtained with *B. tequilensis* (NR104919) followed by another *B. tequilensis* (MN543830). Similarly, for the other isolate TC-4 (ON629736), the maximum similarity was obtained with *B. haynesii*, and for TC-6 (KY575578), it was obtained with *B. subtilis*. Although these results are more reliable as they are based on type material confirming the identity, we did not just rely on the BLASTn results. We further performed phylogenetic analysis and as shown in Figure 1, TC-4 grouped separately with *B. haynesii* isolates, away from the clade containing *B. subtilis* and *B. tequilensis*, with a high bootstrap support of 98–99%. Hence, the identity of the isolates was further confirmed based on the phylogenetic relationship between effective endophytes and type strains’ sequences retrieved from the NCBI database (Figure 1). All these sequences have been deposited at GenBank NCBI, and the pure cultures have been deposited at a designated microbial repository for agriculturally important microorganisms (AIMs) under the National Biodiversity Act, 2002 and a member of World Federation of Culture Collections (WFCC; Table 1).

**Table 1:** Details of the most promising endophytic bacterial bioagents against *Xanthomonas citri* pv. *punicae* causing bacterial blight of pomegranate.

Isolate code	Molecular identity	GenBank accession number	NAIMCC accession number^*^
TC-4	*Bacillus haynesii*	ON629736	NAIMCC-B-03178
TC-6	*Bacillus subtilis*	KY575578	NAIMCC-B-03179
TC-310	*Bacillus tequilensis*	KY575582	NAIMCC-B-03180

^*^National Agriculturally Important Microbial Culture Collection, NAIMCC, Mau, India.

### 3.3 Antimicrobial properties of the potent endophytic bacteria

An antibiosis test was performed using a method that prevented direct contact between the pathogen and endophyte. This means that whatever inhibition was observed in the growth of the pathogen could be due to the volatiles released by the endophytic bacteria. The antibiosis results revealed that the endophytes could inhibit the growth of the pathogen and reduce the production of yellow pigment (Supplementary Figure S1C). Thus, volatile organic compound (VOC) profiling of the isolates was performed using GC–MS. Indeed, GC–MS-based VOC profiles of the isolates (*B. subtilis* and *B. tequilensis*) were found to be composed of some really useful bioactive compounds, such as fatty acids and benzimidazole derivatives, which have reported antimicrobial activities. Some of these compounds also exhibit their anti-bacterial activity by inhibiting biofilm production by pathogens and interfering with quorum sensing of the bacteria (Table 2; Supplementary Figure S2). Hexadecanoic acid methyl ester and Pyrrolo[1,2-a] pyrazine-1,4-dione hexahydro-3-(phenylmethyl) were identified in *B. subtilis* and *B. tequilensis*. N-acetyl-3-methyl-1,4-diazabicyclo [4.3.0] nonan-2,5-dione was identified in *B. tequilensis* only, while 2-(p-(Dimethyl amino) phenyl) benzimidazole and 9,9-dimethyl-Xanthene were identified in *B. subtilis* only.

**Table 2:** List of bioactive compounds identified in VOC profiles of bacterial endophytes.

Volatile organic compound	Retention time (min)	% Area	Chemical formula	Molecular weight (g/mol)	Bioactivity/role/function reported in literature
Hexadecanoic acid methyl ester (palmitic acid)	22.06	15 and 74%	C_17_H_34_O_2_	270.4507	Antimicrobial (Rajaofera et al., 2019)
N-acetyl-3-methyl-1,4-diazabicyclo [4.3.0] nonan-2,5-dione	23.14	3%	C_10_H_14_N_2_O_3_	210.23	Anti-bacterial (Sharma and Thakur, 2020; Awan et al., 2023)
2-(p-(Dimethylamino)phenyl) benzimidazole	23.46	0.08%	C_15_H_15_N_3_	237.31	Anti-fungal (Wright, 1949; Beč et al., 2023)
Pyrrolo[1,2-a] pyrazine-1,4-dione hexahydro-3-(phenylmethyl)	23.91	1.7 and 9%	C_14_H_16_N_2_O_2_	244.29	Antimicrobial: Inhibit bacterial biofilm formation, quorum sensing (Gopi et al., 2014; Kannabiran, 2016; Kiran et al., 2018; Maia et al., 2022)
9,9-dimethyl-Xanthene	21.32	0.02%	C_15_H_14_O	210.27	

### 3.4 Evaluation of host biochemical response upon endophyte inoculation

**Figure 2:**
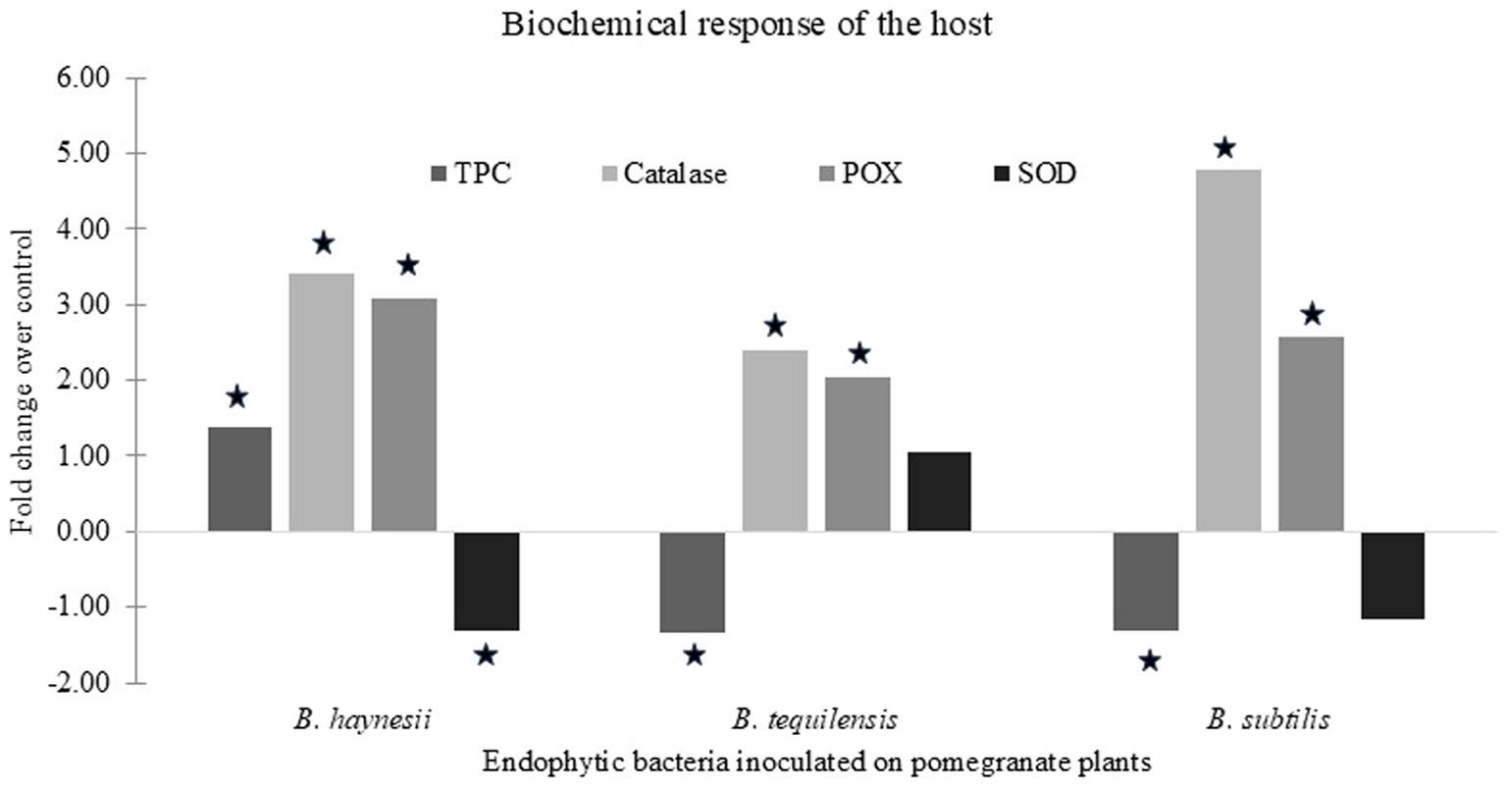
Biochemical response of host upon endophyte inoculation. Fold change in levels of total phenolic content (TPC) and activities of anti-oxidative enzymes such as catalase, peroxidase (POX), and superoxide dismutase (SOD) in pomegranate plants inoculated with endophytes over un-inoculated control plants. Values that are statistically significant (*p* < 0.05) have been indicated by a (star) sign on the bars.

In our previous study, the effect of endophytes on host physiological parameters was studied. Therefore, in the current study, we further evaluated the effect of the exogenous application of endophytes on the anti-oxidant machinery of the host plants (Figure 2). It was found that the effect of endophytes on different enzyme activities varied. *B. haynesii* and *B. subtilis* upregulated catalase and peroxidase activity and downregulated SOD activity, while the trend was reversed in the case of *B. tequilensis*. The accumulation of total phenolics also differed under the influence of these endophytes. *B. haynesii*-treated plants accumulated more phenolics as compared to the control, while *B. subtilis* and *B. tequilensis* treatment caused a reduction in the levels of total phenolics as compared to the control. This reaffirmed that the endophytes exhibited their antagonistic effects via different mechanisms. Hence, the endophytes can be used to form a consortium to be used as a biocontrol agent against Xcp.

### 3.5 Host colonization by the endophytes

To validate any endophyte-mediated response, it is important to understand the colonization pattern. Since all the endophytes altered the host’s biochemical response and host–pathogen interaction, we further validated the colonization of host leaf tissue by the endophytes following artificial inoculation. One of the most effective endophyte *B. tequilensis* (TC-310) was sprayed onto plants and recovered from leaves after periodic isolations. The endophyte could be recovered up to 7 days post-artificial inoculation of endophytes on host plants indicating their successful colonization. Hence, the observed changes in host biochemical response or reduction in blight incidence mediated by the endophytes can be attributed to the successful colonization by the endophytes (Supplementary Figure S3). However, more elaborate studies are required to illustrate colonization traits of the endophytic bacteria.

### 3.6 Bio-efficacy of potent endophytic bacteria under field conditions

**Figure 3:**
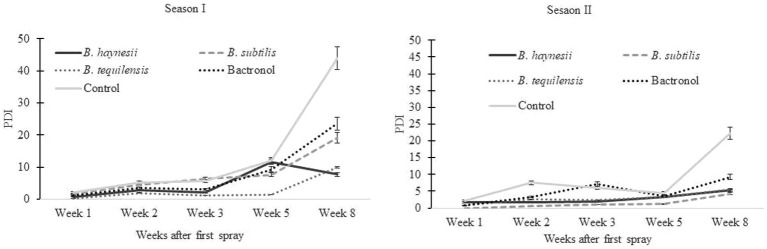
Biocontrol of bacterial blight of pomegranate under field conditions using endophytic bacteria indicating a reduction in disease (PDI: percent disease index) as compared to chemical check [2-bromo-2-nitro-1, 3-propanediol (bactronol)] and un-treated control. **(A)** Trials during the 2022 rainy season crop. **(B)** Trials during the 2023 rainy season crop. Values presented are mean of three replicates, and error bars are based on standard deviation.

The current study reports, for the first time, the successful application of endophytes for biocontrol of bacterial blight in pomegranate under field conditions (Supplementary Figure S4). Endophytic bacterial isolates (*B. haynesii, B. subtilis*, and *B. tequilensis*) were used for field trials against blight, which were conducted during *Mrig* bahar (May–June crop; second or monsoon flowering) for 2 consecutive years (2022 and 2023). The percent disease index (PDI) on pomegranate fruits ranged from 2.2 to 36.4% during season I (2022) and 5.6 to 17.7% during season II (2023) in the control (Figure 3). All the endophytes could reduce the disease in the range of 47 to 68% over the control. Interestingly, this reduction of disease over control was higher than the reduction due to chemical immune modulators in both seasons (Supplementary Table S1). PDI on leaves and stems remained below 1% throughout the trials during both seasons.

### 3.7 Chemical sensitivity of the bacterial endophytes

**Figure 4:**
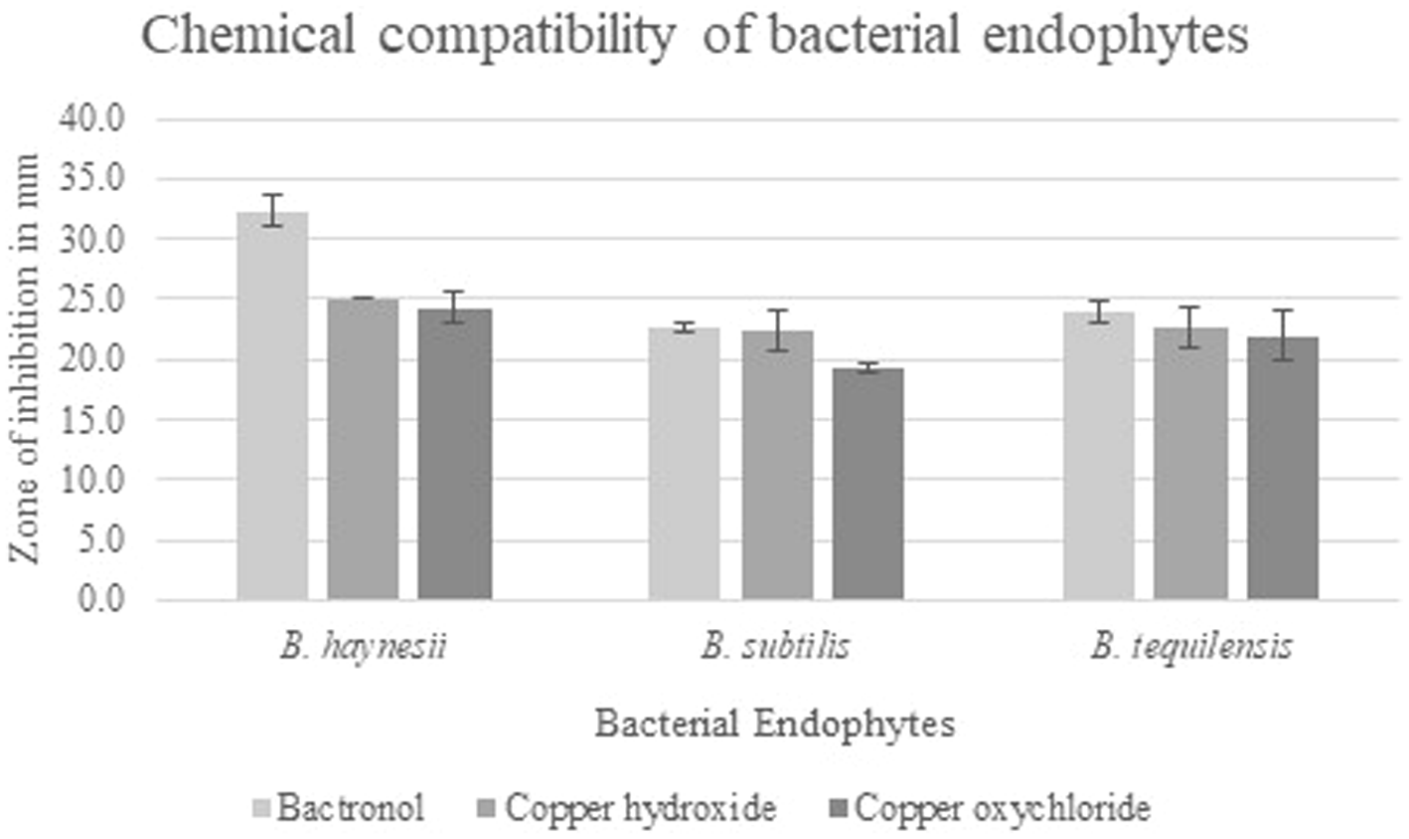
Chemical sensitivity of bacterial endophytes. Growth inhibition in terms of zone of inhibition of three bacterial endophytes *B. haynesii, B. subtilis*, and *B. tequilensis* on chemicals such as 2-bromo-2-nitro-1, 3-propanediol (bactronol), copper hydroxide, and copper oxychloride. Values presented are mean of three replicates, and error bars are based on standard deviation.

All three endophytes showed reduced sensitivity with the chemicals such as immune modulator (2-bromo-2-nitro-1, 3-propanediol) and copper-based compounds (copper hydroxide and copper oxychloride) at their recommended doses (Supplementary Figure S5). *Bacillus subtilis* was found to be the least sensitive to all three chemicals with a maximum inhibition of 23% in 2-bromo-2-nitro-1, 3-propanediol. Similarly, 2-bromo-2-nitro-1, 3-propanediol inhibited the growth of *B. tequilensis* by 24%, and maximum inhibition was found in *B. haynesii* (33%). However, none of the chemicals exhibited growth inhibition of 50%, and hence, the endophytes can be called less sensitive to these chemicals (Figure 4).

## 4 Discussion

*Xanthomonas* genus containing 27 species can infect more than 400 plant hosts and hence is notorious for causing huge economic losses to agriculture and horticulture, worldwide (Ryan et al., 2011). The use of microbial biological control agents (BCAs) for biocontrol of such phytopathogens is one of the eco-friendly and sustainable approaches (Marin et al., 2019). However, in several examples of biocontrol of *Xanthomonas* spp., field studies have not been carried out. Therefore, the current study provides the first-ever successful on-field application of endophytic *Bacillus* spp. to control bacterial blight causing *X. citri* pv*. punicae* (Xcp), a deadly pathogen of pomegranate. The study also provides novel insight into the antimicrobial activity of these endophytic *Bacillus* spp.

Endophytes isolated from micro-propagated pomegranate plants were able to successfully reduce the bacterial blight incidence and severity in pomegranate under polyhouse conditions (Singh et al., 2023). In the current study, the mode of action of these isolates (TC-4, TC-6, and TC-310) that showed maximum inhibition of Xcp was further validated. Based on their genetic relatedness to the type material sequences, these endophytes were identified as *Bacillus haynesii* (TC-4), *B. tequilensis* (TC-310), and *B. subtilis* (TC-6) (Figure 1). While the majority of the studies have reported *B. subtilis* as a successful biocontrol agent, only a few studies have reported *B. tequilensis* as a biocontrol agent and that too mostly against fungal pathogens (Li et al., 2018; Xu et al., 2019; Baard et al., 2023). Very recently, Eltokhy et al. (2024) reported the capability of *B. haynesii* to produce antimicrobial metabolites, which were antagonistic to several multi-drug-resistant pathogens. Nevertheless, to the best of our knowledge, this is the first study where endophytic *B. haynesii* has been reported as a BCA against any bacterial pathogen in plants.

In the current study, the results of the dual plate antibiosis assays suggested that the endophytic *Bacillus* spp. produced volatiles that not only inhibited the growth of the pathogen but also reduced the production of the yellow pigment by the pathogen. The yellow xanthomonadin pigments produced by phytopathogenic *Xanthomonas* spp. are crucial for the epiphytic survival of the pathogen during pathogen–host interaction (Basu and Wallen, 1967; Poplawsky and Chun, 1998; He et al., 2020). Several studies report epiphytic colonization as the first step in *Xanthomonas* infection cycle. While the pathogen remains on the leaf surface epiphytically, it has to protect itself from UV rays (sunlight) and other oxidative stressors. These yellow pigments also provide protection to the bacteria against such oxidative stress (He et al., 2020). Hence, a reduction in pigment production by Xcp, under the influence of *Bacillus* spp. as observed in the current study, indicates reduced survival fitness of the pathogen and compromised anti-oxidative activity.

The endophytic *Bacillus* spp. can have several ways of inhibiting the growth of the target pathogen, including the emission of volatiles (Bruisson et al., 2019) or induction of host defense response (Chandrasekaran and Chun, 2016; Oukala et al., 2021). In the current study, GC–MS-based VOC profiling of the endophytes confirmed the production of several bioactive compounds, by the isolates (Supplementary Figure S2). Additionally, an induction in anti-oxidant enzyme activities along with an increase in phenolic levels (Figure 2) in host cells suggested the possible defense priming of the host by the endophytes. These volatile compounds with known antimicrobial functions included hexadecanoic acid, which has been reported to be synthesized by different species of *Bacillus* and implicated to be an important component of their anti-fungal activity (Rajaofera et al., 2019); however, in the current study, it could be an important component of their anti-bacterial activity against *Xanthomonas*. Another compound found in the profiles of the effective endophytes was N-acetyl-3-methyl-1,4-diazabicyclo [4.3.0] nonan-2,5-dione, which belongs to the piperazine group of compounds and has recently been reported in profiles of a strain of *B. subtilis* that showed anti-fungal activity (Awan et al., 2023). This compound has also been reported as a major component of bioactive profiles of actinobacteria isolated from forest soil (Sharma and Thakur, 2020); however, its direct antimicrobial effects have never been reported earlier. While the antimicrobial activities of other piperazine compounds are known (Jadhav et al., 2017), there is a need to discern the specific antimicrobial effects of N-acetyl-3-methyl-1,4-diazabicyclo [4.3.0] nonan-2,5-dione. A pyrrole derivative, Pyrrolo [1,2-a] pyrazine-1,4-dione hexahydro-3-(phenylmethyl), was observed in the current VOC profiles. This compound has been reported in *Bacillus* spp. (Gopi et al., 2014) and has proven antimicrobial activities mediated by inhibition of quorum sensing of the pathogen (Kannabiran, 2016; Kiran et al., 2018). Similarly, xanthene derivatives also inhibit the growth of the pathogen by disrupting quorum sensing and biofilm synthesis by the pathogen (Maia et al., 2022); however, specific antimicrobial activity for 9,9-dimethyl xanthene (observed in VOC profiles in the current study) has not been reported yet and calls for further research. *Bacillus* VOCs can have other biocidal actions by disturbing the cell physiology and cell membrane integrity as they try to enter the aqueous phase of the cell membrane (Massawe et al., 2018). However, more elaborate studies are required to ascertain the mode of action of these inhibitory volatiles observed in the current study.

Non-phytopathogenic bacteria, such as endophytes, trigger plant immunity by recognizing microbe-associated molecular patterns (MAMPs) through the host cells. This results in a burst of reactive oxygen species (ROS) as a part of the host’s first line of defense (Newman et al., 2013). To overcome this burst and gain entry into plant cells, endophytes induce ROS-scavenging mechanisms (Liu et al., 2017). ROS scavenging could be the mechanism by which the endophyte B. tequilensis colonized pomegranate leaves. This is because the activities of all three enzymes, which are crucial in ROS scavenging, were induced upon inoculation with *B. tequilensis*.

Since there are only a few reports on successful on-field biological control of bacterial blight in pomegranate, the final objective of the study was to ascertain the applicability of endophytes and test their efficacy under field conditions. Many studies report antagonistic activities of biocontrol agents against pathogens *in vitro* or under polyhouse conditions but fail to extend their results to the field. Earlier researchers have used lactic acid bacteria (LAB) for successful integrated management of pomegranate bacterial blight where they reported the effect of LAB to be at par with the antibiotic streptocycline (Gajbhiye et al., 2023). In the current study, the performance of endophytes was better than or at par with the performance of the immune modulator (2-bromo-2-nitro-1, 3-propanediol) under field conditions where a reduction (in disease) of 47–61% during season I and 53 to 68% in season II was observed. Hence, for the first time, the biocontrol of bacterial blight, with the application of endophytes, is reported under field conditions. Under field conditions, it is difficult to say conclusively whether the sprays (5 sprays at 5-day intervals) acted in the prophylactic mode or curative mode, but the observed reduction in disease index could be attributed to the various underlying antagonistic mechanisms employed by *Bacillus* spp. The regular sprays could have primed the defense response of the host plants against Xcp, or the emission of the volatiles by the endophytes could have inhibited the growth and epiphytic survival of the pathogen itself by disrupting biofilm formation. Biofilm formation is an important component in *Xanthomonas* virulence, and strains lacking the ability to form biofilm lose their pathogenicity over the host (Mina et al., 2019).

Moreover, the tested endophytes were less sensitive to chemicals such as 2-bromo-2-nitro-1, 3-propanediol, copper hydroxide, and copper oxychloride at the tested doses. This is noteworthy because once the ban on the antibiotic streptocycline is imposed, these chemicals are the only effective measures to manage bacterial blight in pomegranate. Copper nanoparticles have also been found to be effective in controlling bacterial blight disease in pomegranate (Chikte et al., 2019). Hence, given the lower sensitivity of the bacterial endophytes with these chemicals, the results of the current study support and advocate the use of endophytes, namely, *B. haynesii, B. subtilis*, and *B. tequilensis* for bacterial blight management either alone or as a part of integrated disease management practices. Such an integrated management approach, utilizing biocontrol agents and copper, has been recently reported to manage center rot in onion (Koirala et al., 2024). However, the strain of *B. subtilis* used in that study was sensitive to copper at 250 ppm, while the isolates reported in the current study were compatible with 2,000 ppm of copper compounds. Similarly, the application of fungicide-compatible endophytic *Trichoderma* spp. has also been recommended for the management of rubber leaf fall disease caused by *Corynespora* (Seekham et al., 2024). Hence, it will be worth investigating in the future whether the integration of these biocontrol agents with other chemicals results in better bacterial blight management in pomegranate.

Based on the results of the current study, we conclude that endophytic *B. haynesii*, *B. subtilis*, and *B. tequilensis* control the bacterial blight incidence and severity in the following two ways (i) by priming the plant defense response and (ii) by hampering the infection mechanism of the pathogen. The host plant (pomegranate) behaves in a similar way against any alien microorganisms trying to enter its tissues, whether they are pathogens or useful endophytes. When endophytes are inoculated on the plants, the plants recognize their MAMPs and mount the first line of defense, which is manifested as a burst of ROS. To overcome this ROS burst and gain entry into host cells, endophytes induce the activity of ROS-scavenging enzymes such as catalase, peroxidase, and superoxide dismutase. This induction of the anti-oxidative machinery of the host against beneficial endophytes also facilitates defense priming against a subsequent pathogen attack. On the other hand, the beneficial endophytes also secrete volatiles that reduce the yellow pigments produced by *Xanthomonas.* These pigments are crucial for the epiphytic survival of the pathogen during its infection cycle on the host and also provide protection against oxidative stress; hence, reduction of pigment indicates reduced pathogen survival ability. Some of the volatiles also possess antimicrobial activities, which can directly kill the cells of the pathogen or hamper the infection process via the disruption of biofilm production. However, more elaborate studies are required to evaluate the molecular mechanism behind the induction of host defense response or the endophyte-mediated induced systemic resistance and understand the role of volatiles in inhibiting biofilm production during Xcp–pomegranate interaction.

## Data Availability

The datasets presented in this study can be found in online repositories. The names of the repository/repositories and accession number(s) can be found in the article/Supplementary material.

## Footnotes

^1^ https://ccari.icar.gov.in/wasp2.0/index.php
