# Supplementary material for: Possible biocontrol of bacterial blight in pomegranate using native endophytic Bacillus spp. under field conditions
Source: Front Microbiol. 2024 Dec 11;15:1491124. doi: 10.3389/fmicb.2024.1491124 (PMC11668753; doi:10.3389/fmicb.2024.1491124)
Supplement: Supplementary file 1 [file Table_1.DOCX]

Supplementary Table 1 Percent disease index (PDI) of bacterial blight of pomegranate under field conditions recorded over 8 weeks.

| Treatment | Week 1 | Week 2 | Week 3 | Week 5 | Week 8 | %ROC |
| --- | --- | --- | --- | --- | --- | --- |
|  | Season I (2022) | | | | | |
| *B. haynesii* (TC-4) | 0.7 ± 0.05 | 4.2 ± 1.5 a | 3.4 ± 0.6 b | 10.3 ± 1.0 a | 16.8 ± 0.4 b | 54 |
| *B. subtilis*  (TC-6) | 1.6 ± 0.13 | 3.9 ± 1.0 a | 5.1 ± 0.8 a | 8.7 ± 0.8 bc | 19.2 ± 4.5 b | 47 |
| *B. tequilensis* (TC-310) | 0.3 ± 0.01 | 1.5 ± 0.2 b | 1.7 ± 0.3 c | 2.1 ± 0.4 d | 14.2 ± 0.1 b | 61 |
| 2-Bromo-2-Nitro-1, 3-Propanediol | 1.1 ± 0.10 | 5.0 ± 0.6 a | 5.6 ± 0.4 a | 7.4 ± 0.5 c | 21.4 ± 5.1 b | 41 |
| Control | 2.2 ± 0.18 | 4.3 ± 0.3 a | 4.5 ± 0.5 ab | 9.1 ± 0.8 ab | 36.4 ± 0.4 ba | - |
| Season II (2023) | | | | | | |
| Treatment | Week 1 | Week 2 | Week 3 | Week 5 | Week 8 | %ROC |
| *B. haynesii* (TC-4) | 1.7 ± 0.38 b | 1.6 ± 0.12 c | 3.2 ± 0.36 b | 2.8 ± 0.26 b | 8.4 ± 0.26 b | 53 |
| *B. subtilis*  (TC-6) | 0.04 ± 0.01 d | 1.0 ± 0.22 c | 2.5 ± 0.02 bc | 1.5 ± 0.29 c | 5.7 ± 0.40 c | 68 |
| *B. tequilensis* (TC-310) | 0.7 ± 0.08 c | 3.3 ± 0.45 b | 2.1 ± 0.04 c | 4.2 ± 0.18 a | 7.9 ± 0.34 b | 56 |
| 2-Bromo-2-Nitro-1, 3-Propanediol | 0.7 ± 0.05 c | 3.2 ± 0.33 b | 5.1 ± 0.66 a | 2.8 ± 0.31 b | 8.3 ± 0.45 b | 53 |
| Control | 5.6 ± 0.42 a | 6.1 ± 0.54 a | 4.9 ± 0.51 a | 4.0 ± 0.24 a | 17.7 ± 0.29 a |  |

Values presented are mean of three replicates ± SD.

%ROC: percent reduction over control.

Same alphabets denote that the treatments are not significantly different at p ≤ 0.05
