# Supplementary material for: Possible biocontrol of bacterial blight in pomegranate using native endophytic Bacillus spp. under field conditions
Source: Front Microbiol. 2024 Dec 11;15:1491124. doi: 10.3389/fmicb.2024.1491124 (PMC11668753; doi:10.3389/fmicb.2024.1491124)

Supplementary Figure 2

Chromatogram TC-6 EA 22.11.2021 C:\Users\CICR\Desktop\FAcO\2021\Bacteria\Analysis\TC-6 EA 22.11.2021.qgd

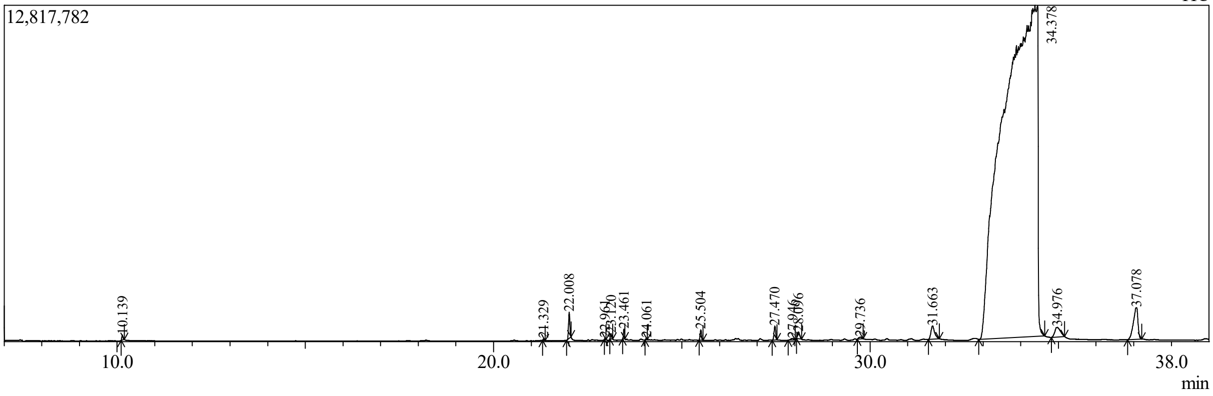

Peak Report TIC

Peak#	R.Time	Area	Area%	Height	Height%	A/H	Name
1	10.139	409388	0.05	189106	1.06	2.16	Phenol
2	21.329	124044	0.02	58418	0.33	2.12	Xanthene, 9,9-dimethyl-
3	22.008	2352943	0.29	985735	5.50	2.39	Azulene, 1,4-dimethyl-7-(1-methylethyl)-
4	22.961	79392	0.01	51778	0.29	1.53	1,2-Benzenedicarboxylic acid, bis(2-methylpropyl) ester
5	23.120	445734	0.05	192667	1.08	2.31	Naphthalene, 1,6-dimethyl-4-(1-methylethyl)-
6	23.461	673714	0.08	364011	2.03	1.85	2-(p-(Dimethylamino)phenyl)benzimidazole
7	24.061	124275	0.02	67313	0.38	1.85	Dibutyl phthalate
8	25.504	961707	0.12	396755	2.22	2.42	2-(p-Methoxyphenyl)-8H-thieno(2,3-b)indole
9	27.470	1620646	0.20	529574	2.96	3.06	1,2-Benzenedicarboxylic acid, butyl 2-ethylhexyl ester
10	27.946	322957	0.04	57105	0.32	5.66	1H-Inden-5-ol, 2,3-dihydro-3-(4-hydroxyphenyl)-1,1,3-tri
11	28.096	1069428	0.13	262957	1.47	4.07	Phenol, 4,4'-(1,3,3-trimethyl-1-propene-1,3-diyl)bis-
12	29.736	349525	0.04	63902	0.36	5.47	N,N-Dimethyldodecanamide
13	31.663	3432846	0.42	506026	2.83	6.78	Diisooctyl phthalate
14	34.378	792242745	96.83	12612269	70.43	62.82	Bis(2-ethylhexyl) phthalate
15	34.976	3773997	0.46	365145	2.04	10.34	Phenol, 2,4-bis(1-phenylethyl)-
16	37.078	10170873	1.24	1204021	6.72	8.45	Phenol, 2,4-bis(1-phenylethyl)-
		818154214	100.00	17906782	100.00		

Spectrum

Line#:1 R.Time:10.140(Scan#:629)
MassPeaks:208
RawMode:Averaged 10.135-10.145(628-630) BasePeak:94(65272)
BG Mode:Calc. from Peak Group 1 - Event 1 Scan

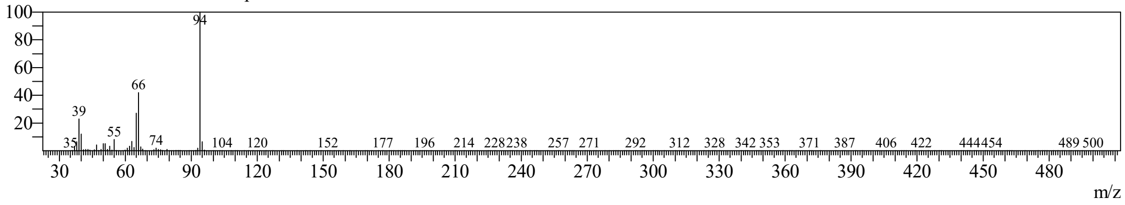

Line#:2   R.Time:21.330(Scan#:2867)
MassPeaks:283
RawMode:Averaged 21.325-21.335(2866-2868) BasePeak:195(21194)
BG Mode:Calc. from Peak Group 1 - Event 1 Scan

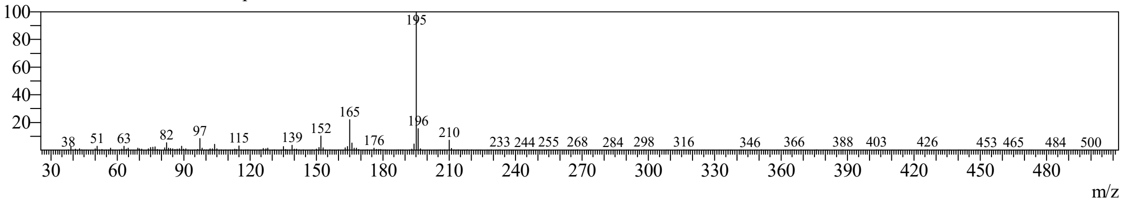

Line#:3   R.Time:22.010(Scan#:3003)
MassPeaks:271
RawMode:Averaged 22.005-22.015(3002-3004) BasePeak:183(208375)
BG Mode:Calc. from Peak Group 1 - Event 1 Scan

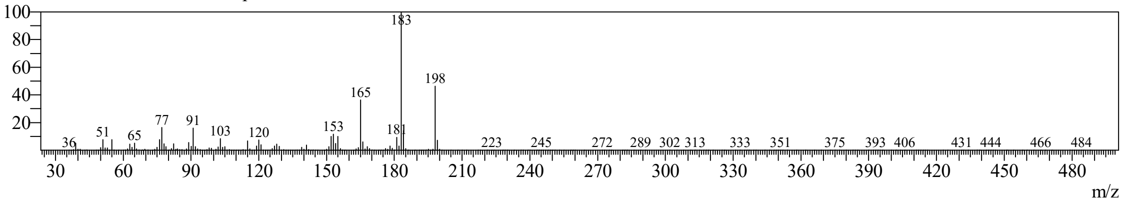

Line#:4   R.Time:22.960(Scan#:3193)
MassPeaks:223
RawMode:Averaged 22.955-22.965(3192-3194) BasePeak:149(24698)
BG Mode:Calc. from Peak Group 1 - Event 1 Scan

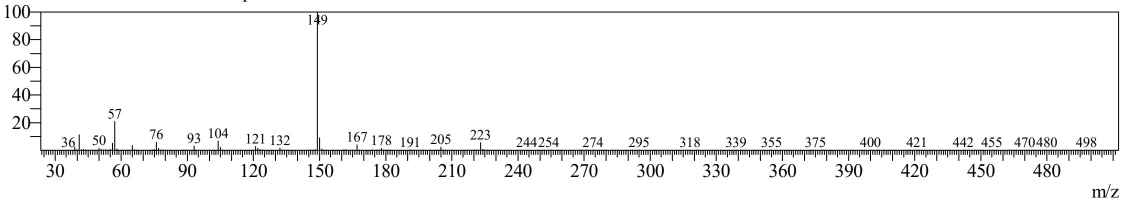

Line#:5   R.Time:23.120(Scan#:3225)
MassPeaks:299
RawMode:Averaged 23.115-23.125(3224-3226) BasePeak:183(44040)
BG Mode:Calc. from Peak Group 1 - Event 1 Scan

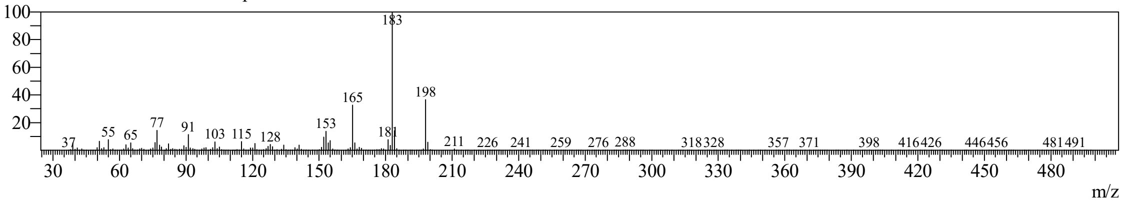

Line#:6   R.Time:23.460(Scan#:3293)
MassPeaks:293
RawMode:Averaged 23.455-23.465(3292-3294) BasePeak:237(132662)
BG Mode:Calc. from Peak Group 1 - Event 1 Scan

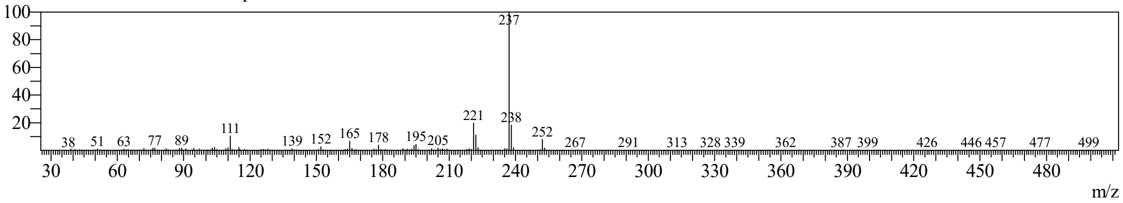

Line#:7   R.Time:24.060(Scan#:3413)
MassPeaks:272
RawMode:Averaged 24.055-24.065(3412-3414) BasePeak:149(34106)
BG Mode:Calc. from Peak Group 1 - Event 1 Scan

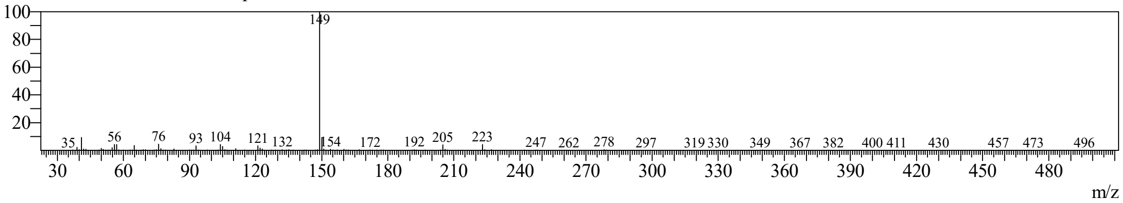

Line#:8 R.Time:25.505(Scan#:3702)
MassPeaks:350
RawMode:Averaged 25.500-25.510(3701-3703) BasePeak:279(150504)
BG Mode:Calc. from Peak Group 1 - Event 1 Scan

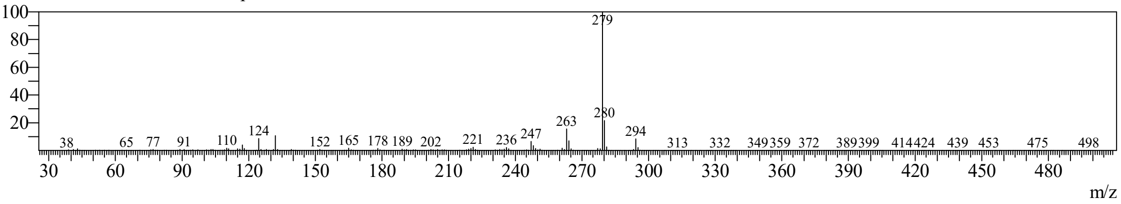

Line#:9 R.Time:27.470(Scan#:4095)
MassPeaks:327
RawMode:Averaged 27.465-27.475(4094-4096) BasePeak:149(227074)
BG Mode:Calc. from Peak Group 1 - Event 1 Scan

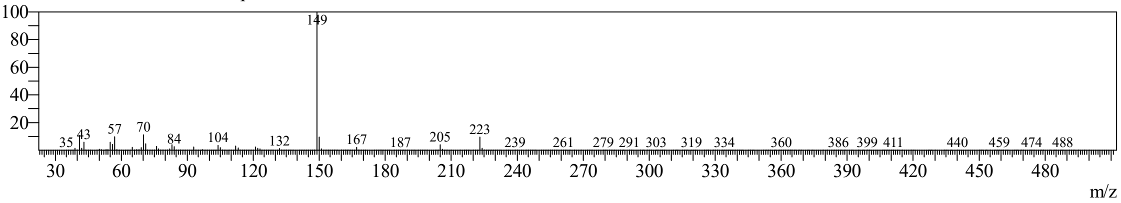

Line#:10 R.Time:27.945(Scan#:4190)
MassPeaks:306
RawMode:Averaged 27.940-27.950(4189-4191) BasePeak:253(5730)
BG Mode:Calc. from Peak Group 1 - Event 1 Scan

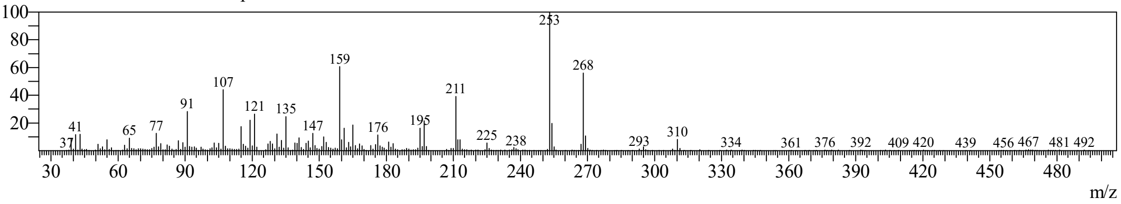

Line#:11 R.Time:28.095(Scan#:4220)
MassPeaks:301
RawMode:Averaged 28.090-28.100(4219-4221) BasePeak:134(28917)
BG Mode:Calc. from Peak Group 1 - Event 1 Scan

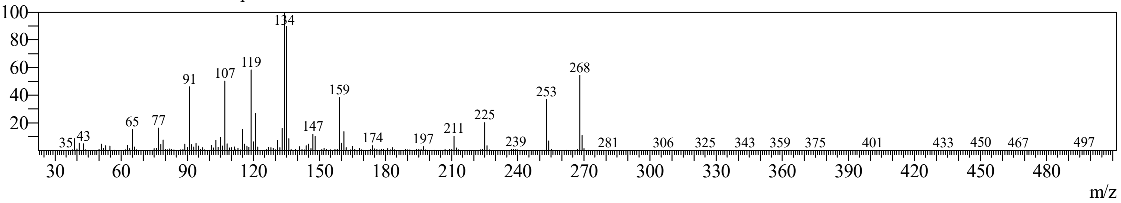

Line#:12 R.Time:29.735(Scan#:4548)
MassPeaks:248
RawMode:Averaged 29.730-29.740(4547-4549) BasePeak:87(23317)
BG Mode:Calc. from Peak Group 1 - Event 1 Scan

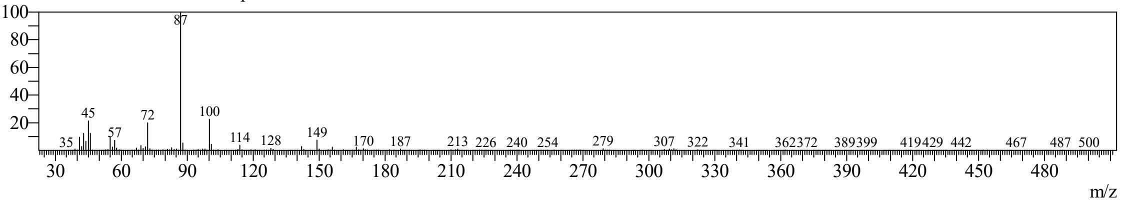

Line#:13 R.Time:31.660(Scan#:4933)
MassPeaks:340
RawMode:Averaged 31.655-31.665(4932-4934) BasePeak:149(118444)
BG Mode:Calc. from Peak Group 1 - Event 1 Scan

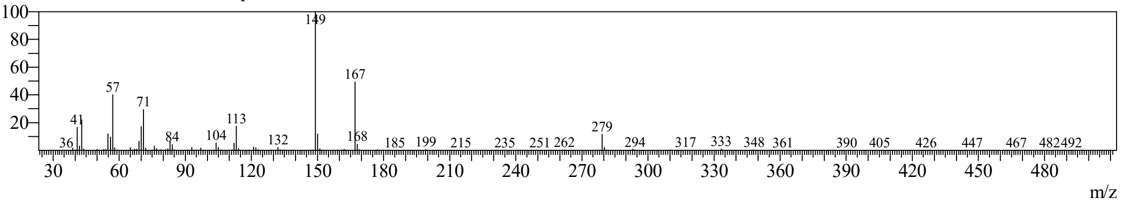

Line#:14 R.Time:34.380(Scan#:5477)
MassPeaks:338
RawMode:Averaged 34.375-34.385(5476-5478) BasePeak:149(3406532)
BG Mode:Calc. from Peak Group 1 - Event 1 Scan

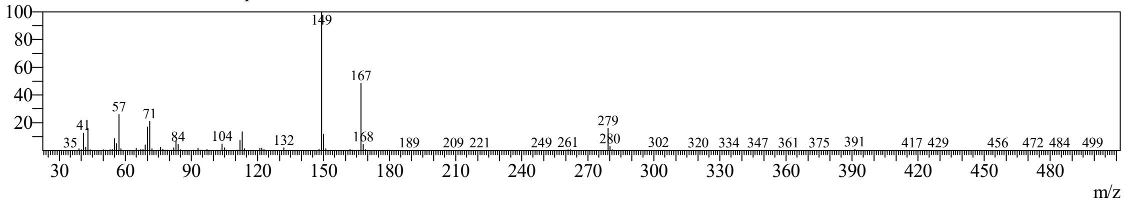

Line#:15 R.Time:34.975(Scan#:5596)
MassPeaks:328
RawMode:Averaged 34.970-34.980(5595-5597) BasePeak:287(49627)
BG Mode:Calc. from Peak Group 1 - Event 1 Scan

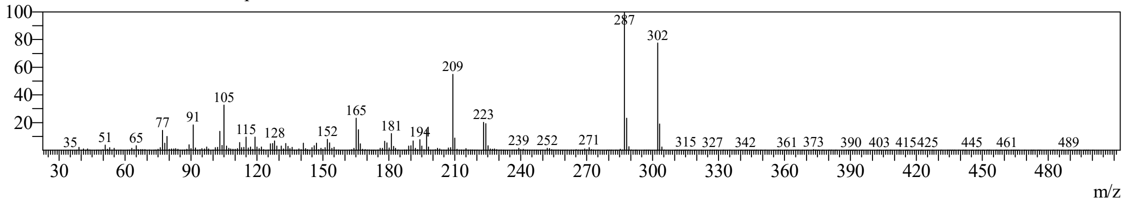

Line#:16 R.Time:37.075(Scan#:6016)
MassPeaks:361
RawMode:Averaged 37.070-37.080(6015-6017) BasePeak:287(229579)
BG Mode:Calc. from Peak Group 1 - Event 1 Scan

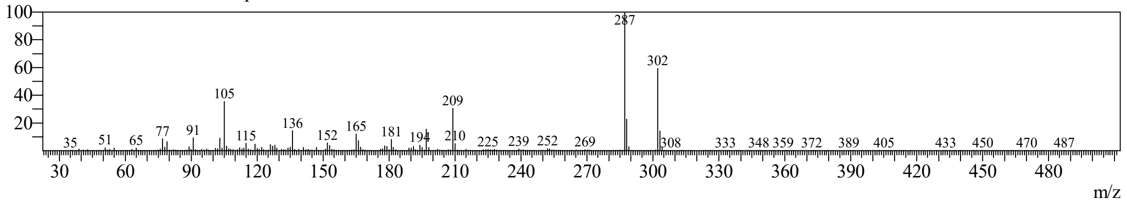

Chromatogram TC-6 methanol 20.11.2021 C:\Users\CICR\Desktop\FAcO\2021\Bacteria\Analysis\TC-6 methanol 20.11.2021.qgd

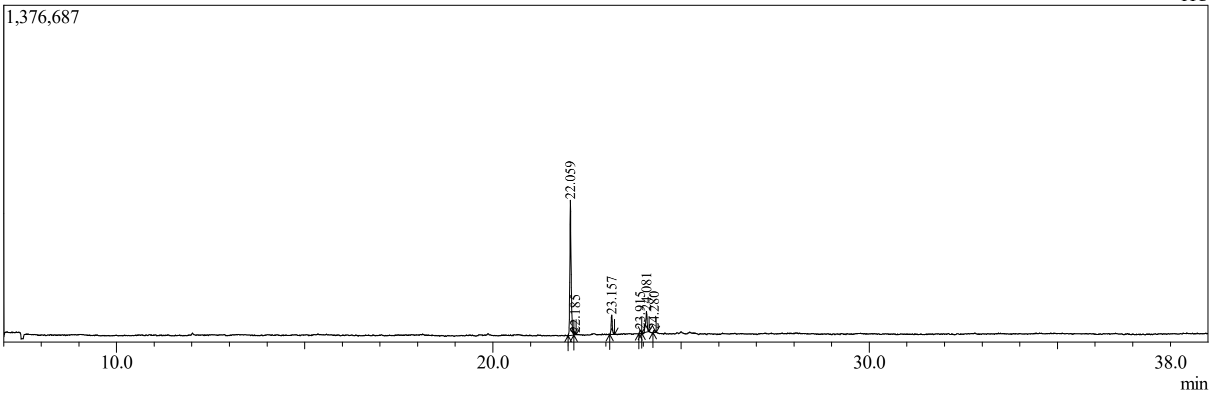

Peak Report TIC

Peak#	R.Time	Area	Area%	Height	Height%	A/H	Name
1	22.059	1387037	74.10	552648	74.42	2.51	Hexadecanoic acid, methyl ester
2	22.185	14540	0.78	4888	0.66	2.97	
3	23.157	178769	9.55	78577	10.58	2.28	Cyclopropaneoctanoic acid, 2-hexyl-, methyl ester
4	23.915	31886	1.70	13237	1.78	2.41	Pyrrolo[1,2-a]pyrazine-1,4-dione, hexahydro-3-(2-methylp
5	24.081	239114	12.77	84813	11.42	2.82	12-Octadecenoic acid, methyl ester
6	24.280	20426	1.09	8479	1.14	2.41	Pyrrolo[1,2-a]pyrazine-1,4-dione, hexahydro-3-(2-methylp
		1871772	100.00	742642	100.00		

Spectrum

Line#:1 R.Time:22.060(Scan#:3013)
MassPeaks:295
RawMode:Averaged 22.055-22.065(3012-3014) BasePeak:74(104734)
BG Mode:Calc. from Peak Group 1 - Event 1 Scan

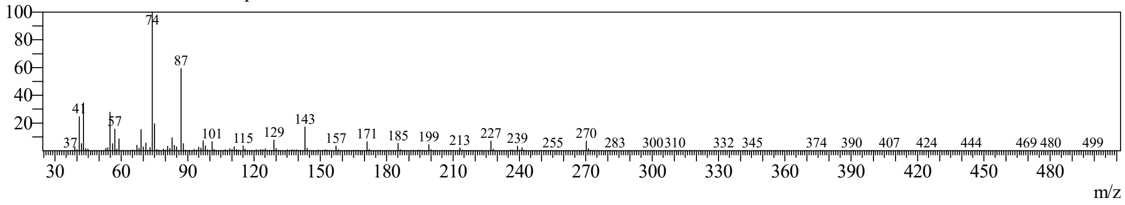

Line#:2 R.Time:22.185(Scan#:3038)
MassPeaks:236
RawMode:Averaged 22.180-22.190(3037-3039) BasePeak:44(114)
BG Mode:Calc. from Peak Group 1 - Event 1 Scan

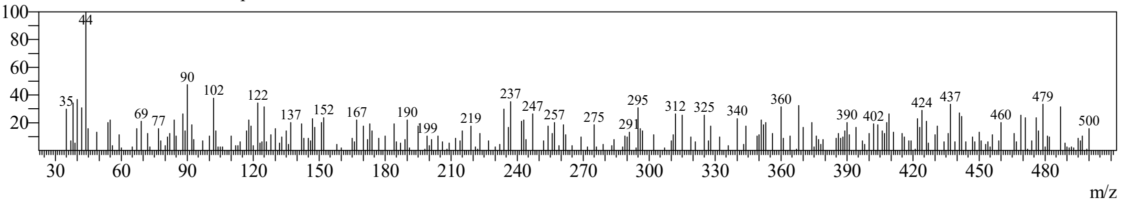

Line#:3 R.Time:23.155(Scan#:3232)
MassPeaks:272
RawMode:Averaged 23.150-23.160(3231-3233) BasePeak:55(6268)
BG Mode:Calc. from Peak Group 1 - Event 1 Scan

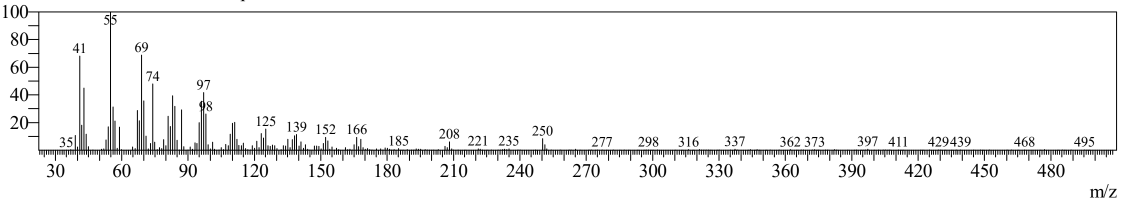

Line#:4 R.Time:23.915(Scan#:3384)
MassPeaks:275
RawMode:Averaged 23.910-23.920(3383-3385) BasePeak:70(2364)
BG Mode:Calc. from Peak Group 1 - Event 1 Scan

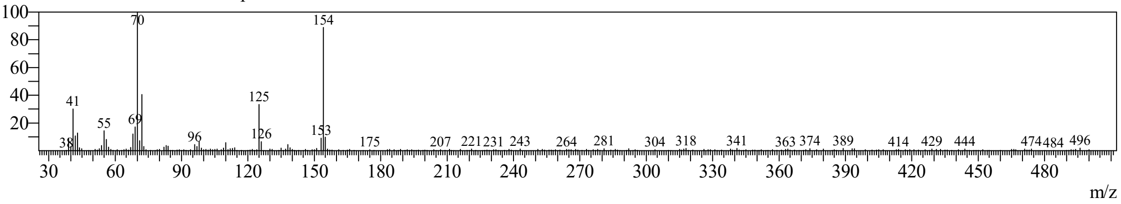

Line#:5 R.Time:24.080(Scan#:3417)
MassPeaks:291
RawMode:Averaged 24.075-24.085(3416-3418) BasePeak:55(6612)
BG Mode:Calc. from Peak Group 1 - Event 1 Scan

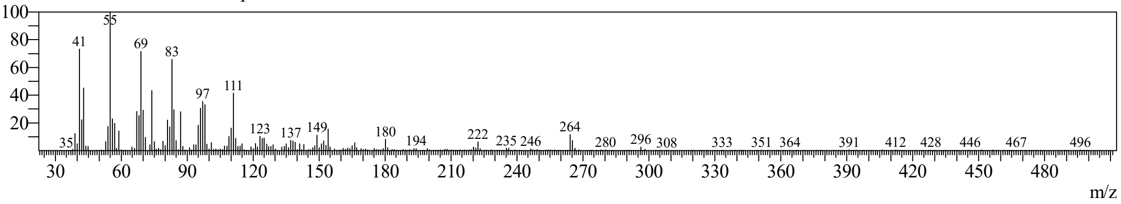

Line#:6 R.Time:24.280(Scan#:3457)
MassPeaks:280
RawMode:Averaged 24.275-24.285(3456-3458) BasePeak:70(1408)
BG Mode:Calc. from Peak Group 1 - Event 1 Scan

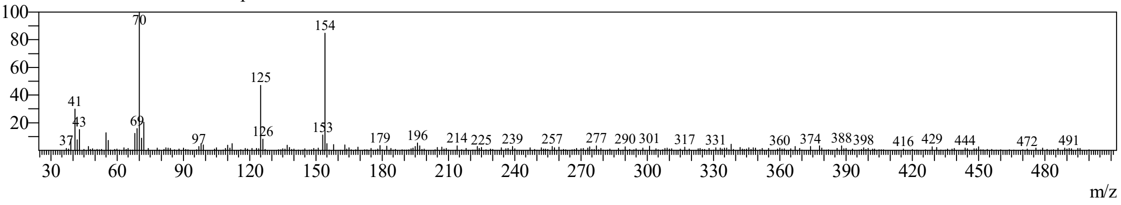

Chromatogram TC-310 methanol repeat 20.11.2021 C:\Users\CICR\Desktop\FAcO\2021\Bacteria\Analysis\TC-310 methanol repeat 20.11.2021.qgd

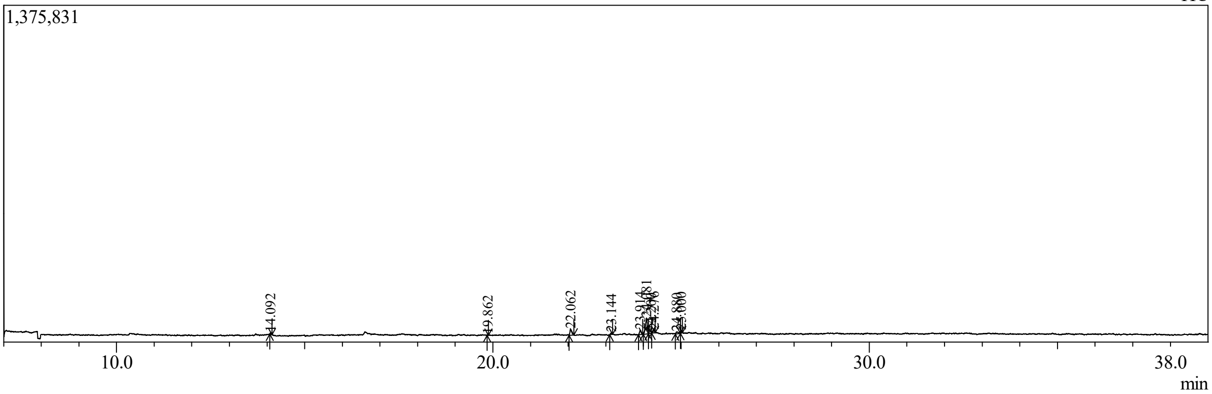

Peak Report TIC

Peak#	R.Time	Area	Area%	Height	Height%	A/H	Name
1	14.092	11446	2.13	6689	4.21	1.71	Naphthalene
2	19.862	3236	0.60	3589	2.26	0.90	RT:19.860
3	22.062	82278	15.31	23978	15.09	3.43	Hexadecanoic acid, methyl ester
4	23.144	17803	3.31	6982	4.39	2.55	3-Methyl-1,4-diazabicyclo[4.3.0]nonan-2,5-dione, N-acet
5	23.914	50516	9.40	17059	10.73	2.96	Pyrrolo[1,2-a]pyrazine-1,4-dione, hexahydro-3-(2-methyl
6	24.081	225426	41.96	64481	40.57	3.50	12-Octadecenoic acid, methyl ester
7	24.200	47608	8.86	6512	4.10	7.31	RT:24.200
8	24.276	35112	6.54	10765	6.77	3.26	Pyrrolo[1,2-a]pyrazine-1,4-dione, hexahydro-3-(2-methyl
9	24.880	40757	7.59	8307	5.23	4.91	
10	25.000	23100	4.30	10564	6.65	2.19	Pyrrolo[1,2-a]pyrazine-1,4-dione, hexahydro-3-(2-methyl
		537282	100.00	158926	100.00		

Spectrum

Line#:1 R.Time:14.090(Scan#:1419)
MassPeaks:266
RawMode:Averaged 14.085-14.095(1418-1420) BasePeak:128(2551)
BG Mode:Calc. from Peak Group 1 - Event 1 Scan

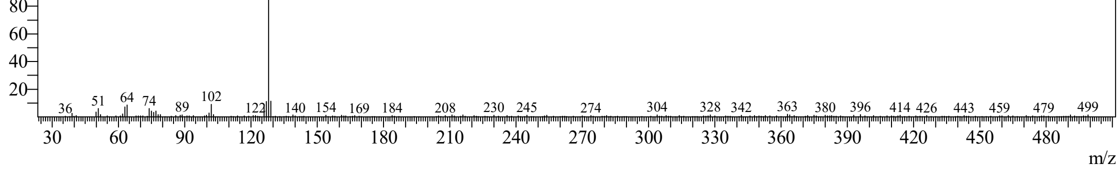

Line#:2 R.Time:19.860(Scan#:2573)
MassPeaks:280
RawMode:Averaged 19.855-19.865(2572-2574) BasePeak:44(54)
BG Mode:Calc. from Peak Group 1 - Event 1 Scan

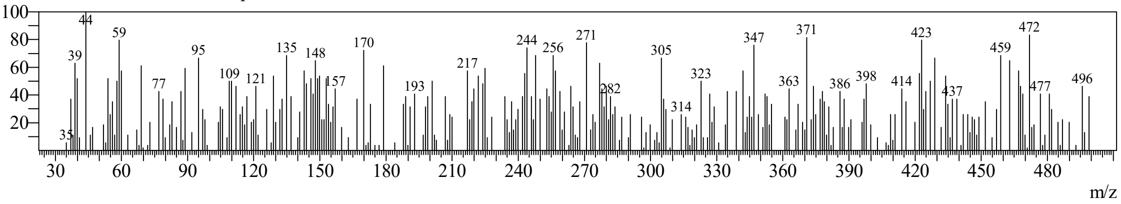

Line#:3 R.Time:22.060(Scan#:3013)
MassPeaks:278
RawMode:Averaged 22.055-22.065(3012-3014) BasePeak:74(4246)
BG Mode:Calc. from Peak Group 1 - Event 1 Scan

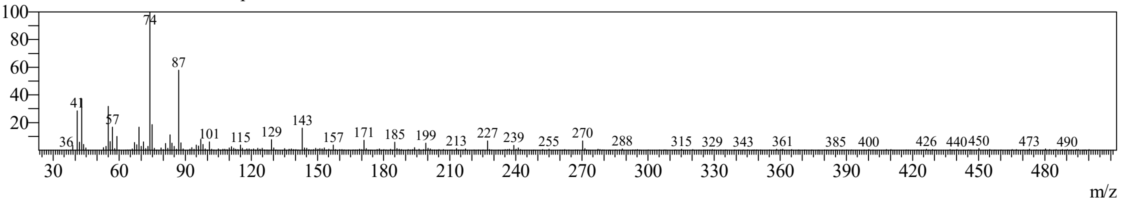

Line#:4 R.Time:23.145(Scan#:3230)
MassPeaks:238
RawMode:Averaged 23.140-23.150(3229-3231) BasePeak:70(1035)
BG Mode:Calc. from Peak Group 1 - Event 1 Scan

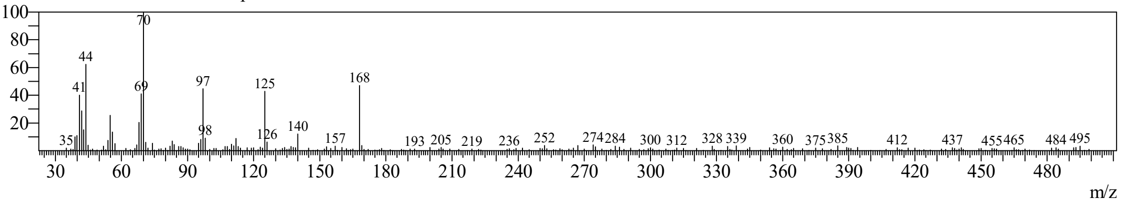

Line#:5 R.Time:23.915(Scan#:3384)
MassPeaks:277
RawMode:Averaged 23.910-23.920(3383-3385) BasePeak:70(3163)
BG Mode:Calc. from Peak Group 1 - Event 1 Scan

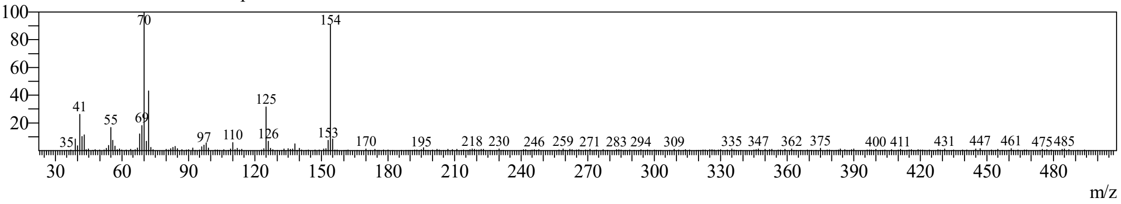

Line#:6 R.Time:24.080(Scan#:3417)
MassPeaks:320
RawMode:Averaged 24.075-24.085(3416-3418) BasePeak:55(4034)
BG Mode:Calc. from Peak Group 1 - Event 1 Scan

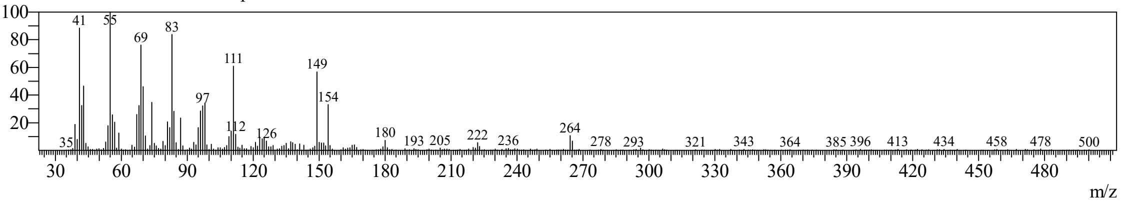

Line#:7 R.Time:24.200(Scan#:3441)
MassPeaks:241
RawMode:Averaged 24.195-24.205(3440-3442) BasePeak:168(252)
BG Mode:Calc. from Peak Group 1 - Event 1 Scan

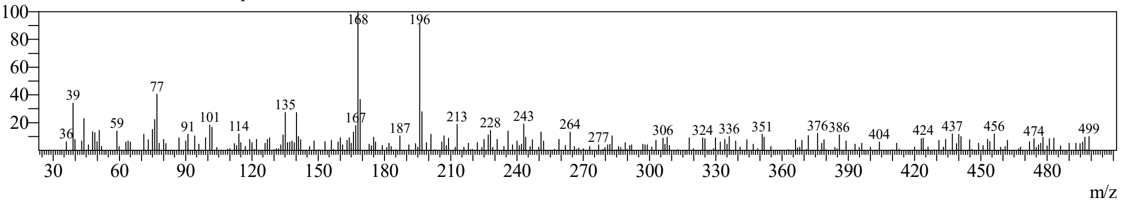

Line#:8   R.Time:24.275(Scan#:3456)
MassPeaks:247
RawMode:Averaged 24.270-24.280(3455-3457) BasePeak:70(1563)
BG Mode:Calc. from Peak Group 1 - Event 1 Scan

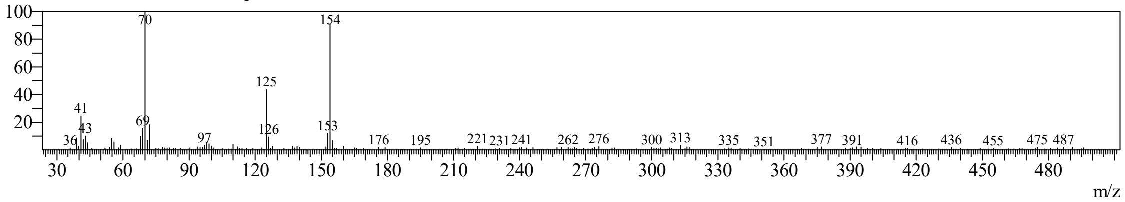

Line#:9   R.Time:24.880(Scan#:3577)
MassPeaks:315
RawMode:Averaged 24.875-24.885(3576-3578) BasePeak:154(733)
BG Mode:Calc. from Peak Group 1 - Event 1 Scan

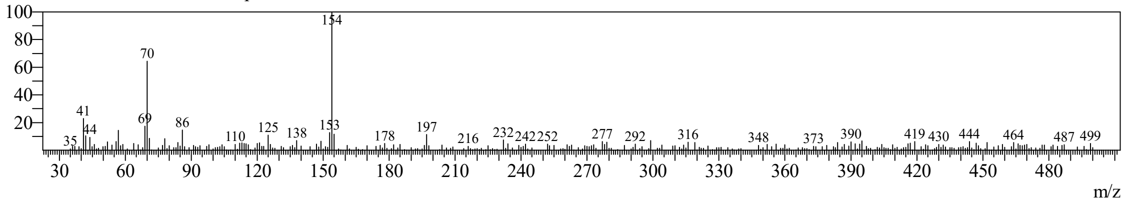

Line#:10   R.Time:25.000(Scan#:3601)
MassPeaks:272
RawMode:Averaged 24.995-25.005(3600-3602) BasePeak:154(1156)
BG Mode:Calc. from Peak Group 1 - Event 1 Scan

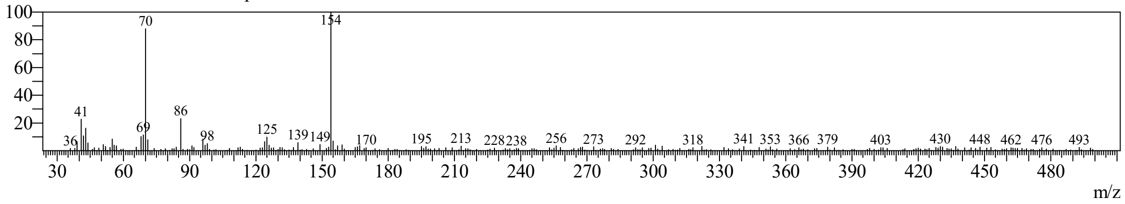

Supplement: Supplementary file 2 [file Data_Sheet_1.zip › Supplementary Figure 2.pdf]
