# Supplementary material for: Possible biocontrol of bacterial blight in pomegranate using native endophytic Bacillus spp. under field conditions
Source: Front Microbiol. 2024 Dec 11;15:1491124. doi: 10.3389/fmicb.2024.1491124 (PMC11668753; doi:10.3389/fmicb.2024.1491124)
Supplement: Supplementary file 2 [file Data_Sheet_1.zip › Supplementary Figure 4.PPTX]

## Slide 1
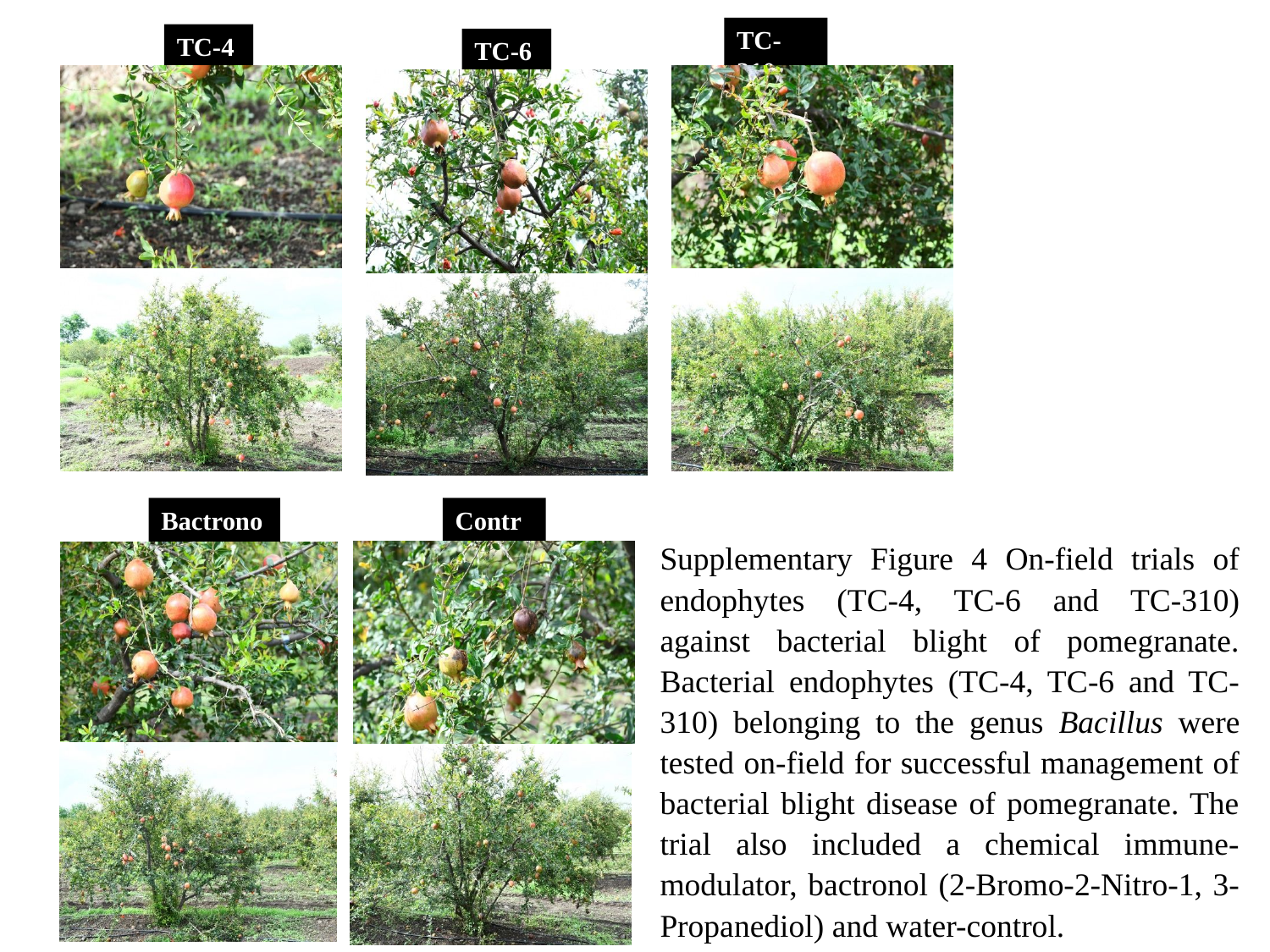

TC-310
TC-4
TC-6
Control
Bactronol
Supplementary Figure 4 On-field trials of endophytes (TC-4, TC-6 and TC-310) against bacterial blight of pomegranate. Bacterial endophytes (TC-4, TC-6 and TC-310) belonging to the genus Bacillus were tested on-field for successful management of bacterial blight disease of pomegranate. The trial also included a chemical immune-modulator, bactronol (2-Bromo-2-Nitro-1, 3-Propanediol) and water-control.
