# Supplementary material for: Possible biocontrol of bacterial blight in pomegranate using native endophytic Bacillus spp. under field conditions
Source: Front Microbiol. 2024 Dec 11;15:1491124. doi: 10.3389/fmicb.2024.1491124 (PMC11668753; doi:10.3389/fmicb.2024.1491124)
Supplement: Supplementary file 2 [file Data_Sheet_1.zip › Supplementary Figure 5.PPTX]

## Slide 1
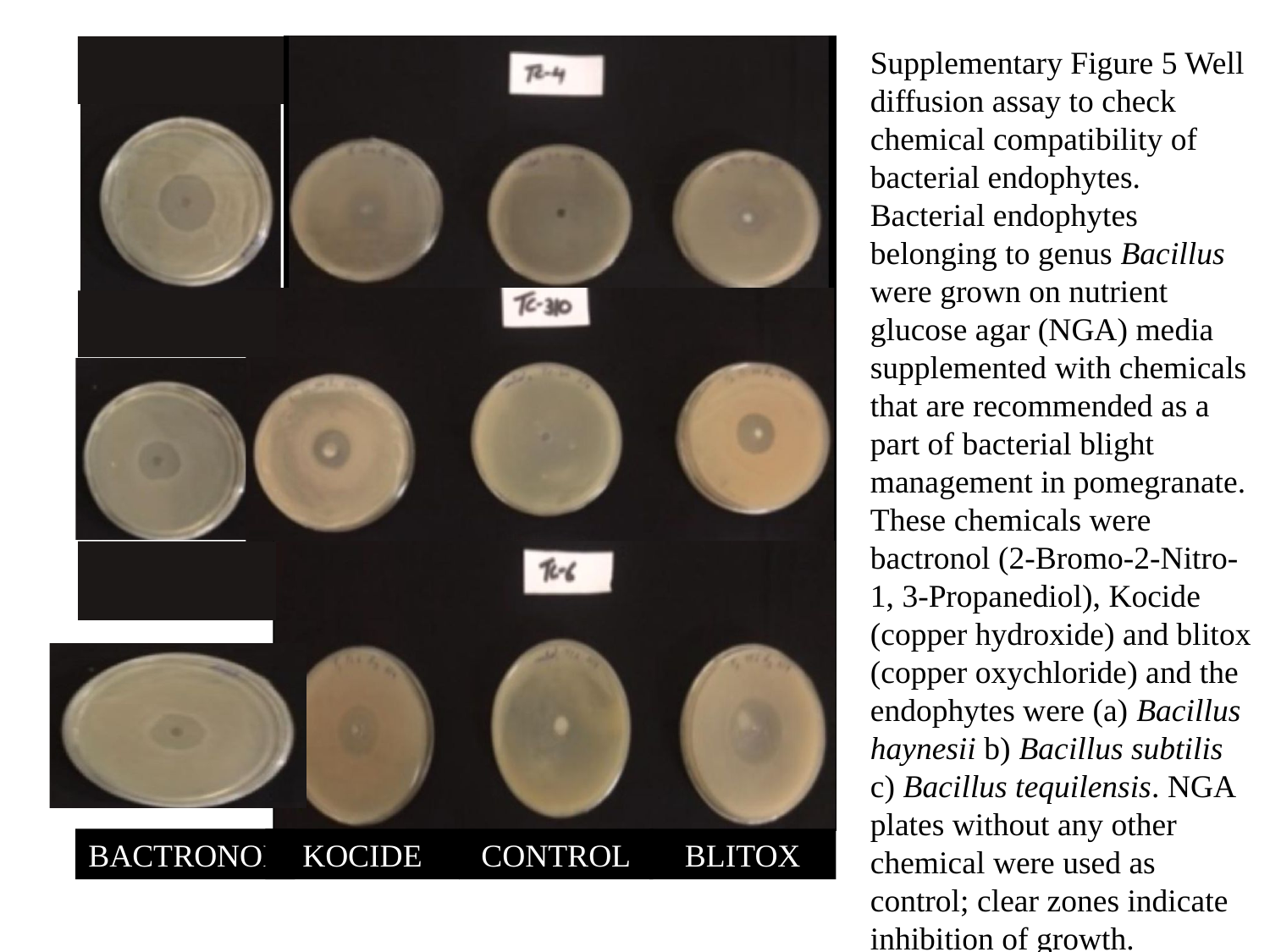

BACTRONOL
KOCIDE
CONTROL
BLITOX
Supplementary Figure 5 Well diffusion assay to check chemical compatibility of bacterial endophytes. Bacterial endophytes belonging to genus Bacillus were grown on nutrient glucose agar (NGA) media supplemented with chemicals that are recommended as a part of bacterial blight management in pomegranate. These chemicals were bactronol (2-Bromo-2-Nitro-1, 3-Propanediol), Kocide (copper hydroxide) and blitox (copper oxychloride) and the endophytes were (a) Bacillus haynesii b) Bacillus subtilis c) Bacillus tequilensis. NGA plates without any other chemical were used as control; clear zones indicate inhibition of growth.
