# Supplementary material for: Possible biocontrol of bacterial blight in pomegranate using native endophytic Bacillus spp. under field conditions
Source: Front Microbiol. 2024 Dec 11;15:1491124. doi: 10.3389/fmicb.2024.1491124 (PMC11668753; doi:10.3389/fmicb.2024.1491124)
Supplement: Supplementary file 2 [file Data_Sheet_1.zip › Supplementary Figure 3.pptx]

## Slide 1
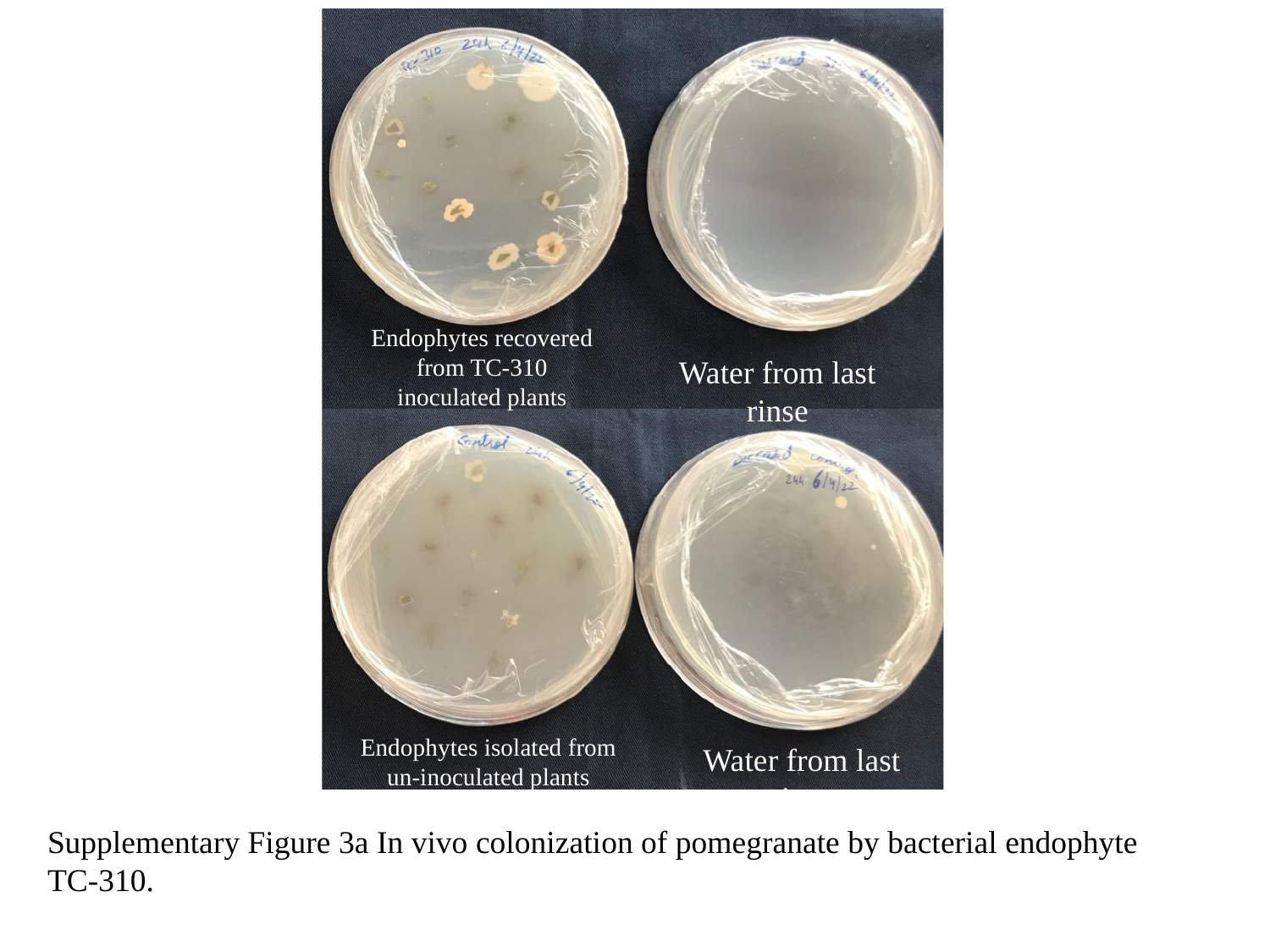

Endophytes recovered from TC-310 inoculated plants
Water from last rinse
Endophytes isolated from un-inoculated plants
Water from last rinse
Supplementary Figure 3a In vivo colonization of pomegranate by bacterial endophyte TC-310.

## Slide 2
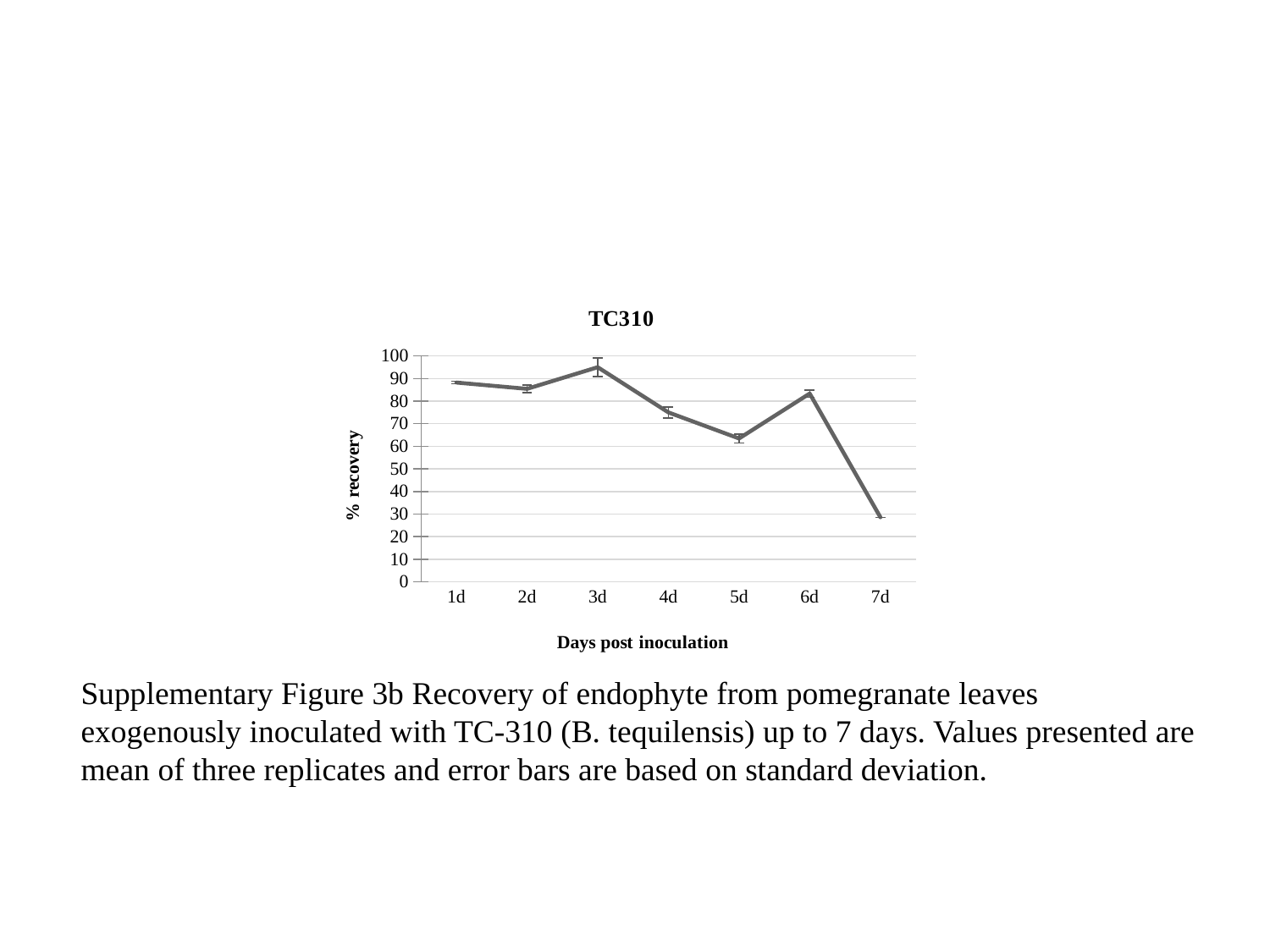

### Chart:
| Category | TC310 |
|---|---|
| 1d | 88.19444444444444 |
| 2d | 85.41666666666667 |
| 3d | 95.0 |
| 4d | 75.0 |
| 5d | 63.46153846153847 |
| 6d | 83.33333333333333 |
| 7d | 28.57142857142857 |Supplementary Figure 3b Recovery of endophyte from pomegranate leaves exogenously inoculated with TC-310 (B. tequilensis) up to 7 days. Values presented are mean of three replicates and error bars are based on standard deviation.
